# Coastal Staphylinidae (Coleoptera): A worldwide checklist, biogeography and natural history

**DOI:** 10.3897/zookeys.107.1651

**Published:** 2011-06-16

**Authors:** J. H. Frank, Kee-Jeong Ahn

**Affiliations:** 1 Entomology and Nematology Department, University of Florida, Gainesville, FL 32611-0630, USA; 2 Department of Biology, Chungnam National University, Daejeon 305-764, Republic of Korea

**Keywords:** seashore Staphylinidae, marine Staphylinidae, littoral Staphylinidae, intertidal Staphylinidae, habitat, behavior

## Abstract

We provide a list of the 392 described species of Staphylinidae confined to coastal habitats worldwide. The list is in taxonomic sequence by subfamily, tribe, and genus and includes 91 genera. We provide the page reference of the original description of every species and genus listed and of many synonyms. We note the existence of recent reviews, phylogenies and keys of each of the tribes and genera included. Coastal Staphylinidae contain eight subfamilies: Microsilphinae, Omaliinae, Pselaphinae, Aleocharinae, Oxytelinae, Scydmaeninae, Paederinae, and Staphylininae.

By ‘coastal habitats’ we mean habitats existing on the sea coast and subject to inundation or at least splashing by the very highest tides. This includes rocky, boulder, coral, sandy, and muddy seashores, and at least portions of salt-marshes, estuaries, and mangrove swamps. We exclude the sand dune habitat and higher parts of sea-cliffs.

The list notes distribution of all the species, first according to the ocean or sea on whose shores it has been recorded, and second by country (and for the larger countries by province or state). Although this distribution is undoubtedly incomplete, it provides a basis for future development of a dedicated database.

The ‘Habitats, Habits, and Classificatory Notes’ section is designed to provide ecologists with further taxonomic and ecological information. It includes references to descriptions of the immature stages, behavior of adults and immatures, their food, natural enemies, and habitat. We would have preferred to separate these entities, but current knowledge of ecology is developed in few instances beyond natural history.

The Pacific Ocean basin was the origin and contributed to the dispersal of the majority of specialist coastal Staphylinidae at the level of genus. However, at the level of species, species belonging to non-coastal-specialist genera are about as likely to occur on the shores of other oceans as on the shores of the Pacific. This difference is a reflection of the antiquity of coastal genera and species.

A complete bibliography, and habitat and habitus photographs of some representative coastal Staphylinidae species are provided.

## Introduction

We struggled to find an appropriate title for this work, but eventually rejected the expressions intertidal, marine, littoral, and seashore, all of which have been used by other authors. By “coastal” we mean species that dwell on sea coasts and are restricted to such habitats. However, we restrict the definition to habitats to those that are normally or occasionally inundated by tides, excluding cliff and dune habitats, as well as inland salt-laden habitats. Thus, those species included dwell in the intertidal zone and at the drift line, and in mangrove swamps, salt marshes, and estuaries where they may be inundated by the tides. We do not employ the terms halophile and halobiont because they refer to organisms that dwell in salt-laden habitats, which are not restricted to coastal areas; indeed saline lakes and ponds occur hundreds of kilometers from coasts, and we do not wish to consider these.

A book chapter on intertidal Staphylinidae (Moore and Legner 1976) was undoubtedly Ian Moore’s major contribution to this, his favorite, subject. Moore had already published numerous papers on intertidal Staphylinidae of the Pacific coast of North America. In this pioneering treatment he endeavored to summarize the world literature on intertidal staphylinids. To do so it was necessary to separate the literature on intertidal species from that on non-marine species with which it was intermixed. The task was daunting because of the number of species in the family (over 54,000 are now recognized), a huge polyglot literature, and the lack of habitat information in many of the early taxonomic publications. He included keys to identification of adults to the level of genus.

In the subsequent 35 years, some genera that Moore dealt with have been revised (see particularly studies by Ahn, Ashe, Assing, Gusarov, Haghebaert, Herman, Jeon, Klimaszewski, Maruyama, and Zerche), additional species have been described, synonymies have been reported, there has been much change in the higher classification of Staphylinidae, and there have been some studies of the behavior of intertidal Staphylinidae. These changes make an updated contribution worth undertaking. This contribution is not simply an updating of Moore and Legner (1976), but it additionally lists all the staphylinid species (not just genera) that are believed to be restricted to coastal habitats. This list is augmented by page-references to their original descriptions in the literature, to references to generic revisions, and to publications on behavior of the species in question. We believe this will enable interested readers to access the original literature more readily. In this contribution, however, we do not include keys to identification of adults. For these the reader is urged to consult the cited literature. This contribution is intended for the reader who is willing and able to tackle the taxonomic literature, even if the ultimate objective is ecological or ethological.

This contribution lists some 392 species, in 91 genera, of Staphylinidae that are believed to be confined to coastal habitats. Some genera are confined to coastal habitats. Others include species that are confined to coastal habitats (and primarily only those with such restricted habitat are included in this treatment). One large genus, *Bledius* Leach, is exceptional in that its members live on banks of either freshwater or saline water bodies. Among the latter group it is in many instances unclear whether they are restricted to marine saline habitats.

Moore and Legner (1976) included not only genera that restricted to intertidal habitats, but also genera whose members had frequently or occasionally been found there. Our contribution is more selective in that it tries to admit only those species for which there is evidence of restriction to such habitats. This attempt to concentrate on true coastal species is emphasized by Smetana (2009) in his critical review of a paper published by Majka et al. (2008). Smetana’s (2009) viewpoint is to exclude those species that are occasional or even frequent visitors to the coastal habitat, and to concentrate on those that are confined to the habitat. There is some difficulty in treating members of the large genus *Bledius* becausesome species occupy not only coastal habitats, but also inland saline habitats; we attempt to exclude species that are not restricted to coastal habitats.

The current epitome of a study of regional coastal staphylinids is that by Hammond (2000). It discusses systematics and distribution of the British species, but it includes species that are not restricted to this habitat. The converse is perhaps Hasegawa and Kanie (1992) which provided a list of Staphylinidae collected at one seashore locality in a 2-year time period, making no distinction between those species restricted to seashores and those incidentally found there, and makes no mention of their wider distribution. Lengerken (1929) published an extensive compilation of the distribution of coastal species of the North, Baltic, Irish, Mediterranean, Black, and Caspian seas. Audisio and Taglianti (2010) presented a compilation of Coleoptera found on Italian coasts, but did not indicate which ones among the included species are restricted to such habitats.

The adjective halophilous seems first to have been used in English in the late 19th century to mean plants that are salt-loving, or growing in salt marshes (OED 1971). Since then, a set of terms evolved to describe adaptations of organisms to saline environments:

1. Halobionts (obligate inhabitants of saline habitats),

2. Halophiles (facultative inhabitants of saline habitats),

3. Haloxenes (halotolerant species),

4. Incidentals (species not specifically associated with saline habitats but regularly found there.

Such terms (in German) were used by Lengerken (1929) and other authors in English and French. We do not use these terms because they do not describe exactly what we want to include (and exclude), which is species that are restricted to sea coasts. We suppose that all of the species we list are halobionts, but we exclude halobionts living on the shores of inland saline lakes.

### The checklist

**Figure 1.**
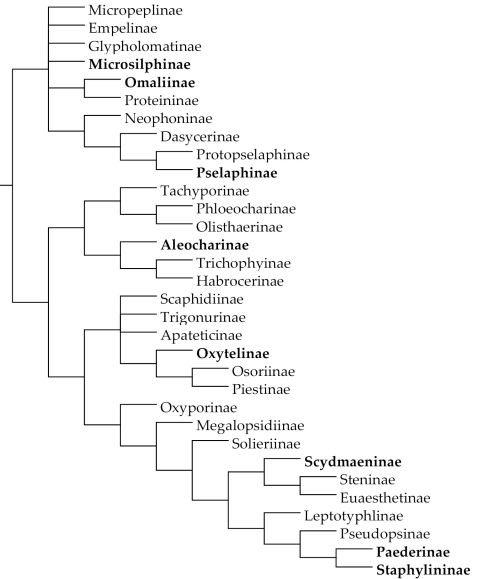
Phylogeny of the Staphylinidae. Bold indicates eight subfamilies containing coastal species. Modified from Newton and Thayer (2007) and Grebennikov and Newton (2009).

This checklist is the first to attempt to enumerate all coastal staphylinids, and their distribution. Arrangement is taxonomic including subfamily, tribe, and genus; subtribes are included where defined. References to original generic and specific description are given. Generic and species synonyms are listed, each with original bibliographic reference. Listing of names of species within genera, genera within tribes, and tribes within subfamilies are mostly alphabetical, but names of subfamilies are arranged in taxonomic sequence. The arrangement followed for the higher categories is that of Lawrence and Newton (2000), Newton and Thayer (2007), and Grebennikov and Newton (2009) (Fig. 1).

To the far right of the taxonomic entries for genera (and in one instance for a tribe) reference to recent taxonomic revisions and phylogenies and keys to identification of adults [e.g., rev. Ahn 1996a; phy. Ahn and Ashe 1996b; key Moore and Legner 1976] is given.

Because this work deals with coastal species, primary geographical entries are given according to the oceans and seas on which species are found. Secondary entries are the names of the countries they inhabit, and tertiary entries (if any) are the (mainly political) subunits of larger countries, or islands belonging to the former. Compression of this information into a checklist required the use of abbreviations, which are as follows:

**A.** Codes used for oceans have 3 letters: ACO (Arctic Ocean), INO (Indian Ocean), NAO (North Atlantic Ocean), NPO (North Pacific Ocean), SAO (South Atlantic Ocean), and SPO (South Pacific Ocean). Names of seas and gulfs are spelled out: Andaman Sea, Arabian Sea, Arafura Sea, Bali Sea, Baltic Sea, Bering Sea, Bismarck Sea, Black Sea, Caribbean Sea, Celebes Sea, East China Sea, East Sea [sometimes called Sea of Japan, but that name is disputed (Wikipedia 2010)], Gulf of California (sometimes called Sea of Cortez), Gulf of Mexico, Irish Sea, Java Sea, Mediterranean Sea (here including Adriatic, Aegean, Ionian, Ligurian and Tyrrhenian Seas), North Sea, Sea of Okhotsk, Philippine Sea, Red Sea, South China Sea, Sulu Sea, Tasman Sea, and Timor Sea.

Names of seas are not used throughout. We have used the name of the ocean in the broad sense (of which the sea is part) in instances where the name of the sea is not apparent from the literature. For example, some species known from New Zealand may be known from the west coast (the Tasman Sea), but if that was not apparent from the literature, we ascribed them to SPO (the South Pacific Ocean).

**B.** Country codes have 2 letters and are the International Standards Organization (ISO) abbreviations. They are given in parentheses. Those used are: AG=Antigua and Barbuda, AL=Albania, AR=Argentina, AU=Australia, BB=Barbados, BE=Belgium, BG=Bulgaria, BM=Bermuda, BR=Brazil, BS=Bahamas, CA=Canada, CL=Chile, CN=China, CO=Columbia, CU=Cuba, CY=Cyprus, DE=Germany, DJ=Djibouti, DK=Denmark, DM=Dominica, DO=Dominican Republic, DZ=Algeria, EC=Ecuador, EE=Estonia, EG=Egypt, ER=Eritrea, ES=Spain, ET=Ethiopia, FI=Finland, FJ=Fiji, FP=French Polynesia, FR=France, GB=Great Britain, GD=Grenada, GE=Georgia, GH=Ghana, GL=Greenland, GP=Guadeloupe, GR=Greece, HR=Croatia, HT=Haiti, ID=Indonesia, IE=Ireland, IL=Israel, IN=India, IQ=Iraq, IS=Iceland, IT=Italy, JM=Jamaica, JP=Japan, KE=Kenya, KN=St. Kitts Nevis, KP=North Korea, KR=South Korea, KY=Cayman Island, LB=Lebanon, LC=St. Lucia, LK=Sri Lanka, LY=Libya, MA=Morocco, MG=Madagascar, MM=Myanmar, MR=Mauritania, MS=Montserrat, MT=Malta, MU=Mauritius, MX=Mexico, MY=Malaysia, NA=Namibia, NC=New Caledonia, NG=Nigeria, NL=Netherlands, NO=Norway, NZ=New Zealand, PE=Peru, PG=Papua New Guinea, PH=Philippines, PL=Poland, PR=Puerto Rico, PT=Portugal, RE=Reunion, RO=Romania, RU=Russian Federation, SA=Saudi Arabia, SC=Seychelles, SD=Sudan, SG=Singapore, SN=Senegal, SE=Sweden, SO=Somalia, TH=Thailand, TN=Tunisia, TR=Turkey, TT=Trinidad and Tobago, TW=Taiwan, TZ=Tanzania, UA=Ukraine, UK=United Kingdom, US=USA, UY=Uruguay, VE=Venezuela, VI=US Virgin Islands, VN=Vietnam, WS=Samoa (formerly Western Samoa, not American Samoa), YE=Yemen, YU=former Yugoslavia, ZA=South Africa.

**C.** Where places within countries are mentioned, they are given after a colon (:) following the abbreviation of the country name, and either are spelled out or are abbreviated. For the USA and Canada, the abbreviations are the 2-letter postal codes (BC=British Columbia, NB=New Brunswick, NL=Newfoundland and Labrador, NS=Nova Scotia, NT=Northwest Territories, PE=Prince Edward Island, QC=Quebec, YT=Yukon Territory); for Mexico they have 2 letters (BN=Baja California, BS=Baja California Sur, CA=Campeche, CH=Chiapas, GU=Guerrero, JA=Jalisco, MI=Michoacán, NA=Nayarit, OA=Oaxaca, QR=Quintana Roo, SI=Sinaloa, SO=Sonora, TB=Tabasco, TM=Tamaulipas, and VC=Veracruz); for Japan, designations are for major islands and island groups: (HK=Hokkaido, HN=Honshu, KY=Kyushu, RY=Ryukyu, SH=Shikoku). For Great Britain (GB) they are England, N. Ireland, Scotland, and Wales.

Compilation of the checklist is a first step in mapping of the distribution of all species. It draws upon information given in the original species descriptions as well as later sources that are believed reliable but are not cited in the bibliography. An ideal compilation would be an online database with a map for each species compiled from published collection records and linked directly to a bibliographic entry.

### Habits and habitats

A checklist by itself reveals nothing about how the insects live. The ultimate in autecology is the numerical assessment of the dynamics of populations. An intermediate step is the study of habitat and behavior, by which can be learned the kinds of limitations to population size. This kind of information is sketchy for most coastal staphylinids, and is hard to present in tabular form. Therefore, this section presents textual information for genera and species whose habitat and behavior are known. Although this information is fundamental to population dynamics, it is also useful for the purposes of zoogeography.

Works on seashores and their fauna at large have traditionally been published by marine biologists. If we wanted to learn about the identification of the fauna on European shores, we might consult Barrett and Yonge (1958) or its replacement (Hayward et al. 1996), but we would be disappointed in coverage of Staphylinidae. If we wanted to read about sandy beaches and their fauna, we might consult McLachlan and Brown (2006), or Little et al. (2009) for rocky shores, or Hogarth (2007) for mangroves and seagrasses. Although we might learn much about those environments, we would again be disappointed in coverage of Staphylinidae. Not until we consult Cheng (1976) for marine insects do we gain an appreciation that many staphylinid species dwell on seashores (the included chapter by Moore and Legner), or Morris et al. (1980) for intertidal invertebrates of California (the included chapter by Evans), or Sherwood et al. (2000) for British coastal invertebrates (the included chapter by Hammond). The fault, if there is one, is the slowness in development of comprehensive treatment by entomologists of coastal staphylinids, and that the groundwork (taxonomic and behavioral studies) may not be in publications that marine biologists usually consult.

**Figure 2.**
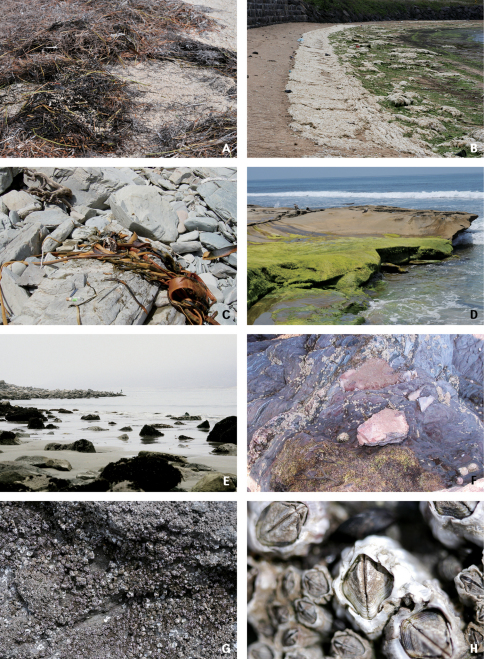
Habitats of coastal Staphylinidae. **A** Seagrasses on a sandy beach in Florida, USA **B** Seaweeds on a sandy beach in Jejudo Island, Korea **C** Kelp on a rocky shore in Greymouth, New Zealand **D** Rocky shore in San Diego, USA **E** Seaweeds on rocks of a sandy beach in California, USA **F** Rock crevice on a rocky shore in Plymouth, England **G** Rock covered with barnacles in Baeksu, Korea **H** Close-up of barnacles.

**Figure 3.**
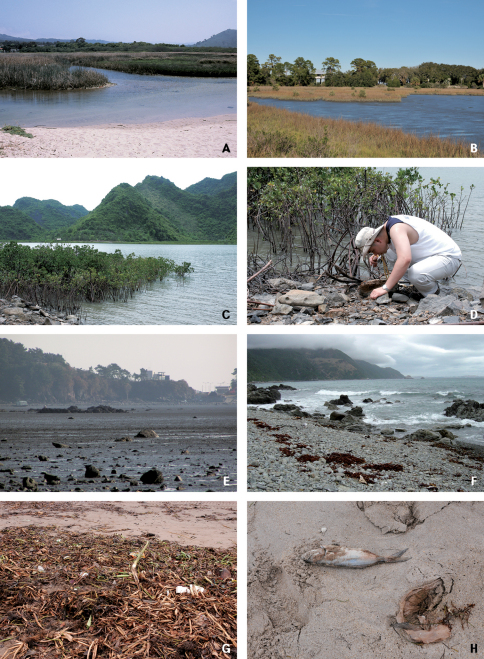
Habitats of coastal Staphylinidae. **A** Estuary of Carmel River in California, USA **B** Salt marsh in Florida, USA **C** Mangrove forests in Cat Ba National Park, Vietnam **D** Under stones of mangrove in Cat Ba National Park, Vietnam **E** Mud flat in Gungpyongri, Korea **F** Pebbles and rocks on beach in Kaikoura, New Zealand **G** Seagrasses on a sandy beach in Haiphong, Vietnam **H** Dead fishes on a sandy beach in Florida, USA.

**Figure 4.**
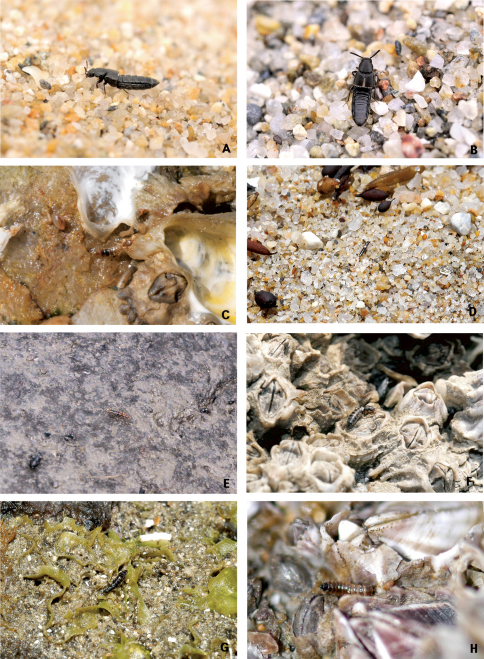
Coastal Staphylinidae. **A** *Aleochara zerchei* on a sandy beach in Donghae, Korea **B** *Aleochara puetzi* on a sandy beach in Donghae, Korea **C** *Bryothinusa koreana* under a stone on rocky headland in Dangjin, Korea **D** *Atheta tokiokai* on a sandy beach in Jejudo Island, Korea **E** *Paramblopusa borealis* under a stone on pebble beach in Alaska, USA **F** *Diaulota aokii* with barnacles on a rocky shore in Baeksu, Korea **G** *Diaulota aokii* with fresh seaweeds on a rocky shore in Jejudo Island, Korea **H** Larva of *Diaulota aokii* with barnacles on a rocky shore in Baeksu, Korea.

**Figure 5.**
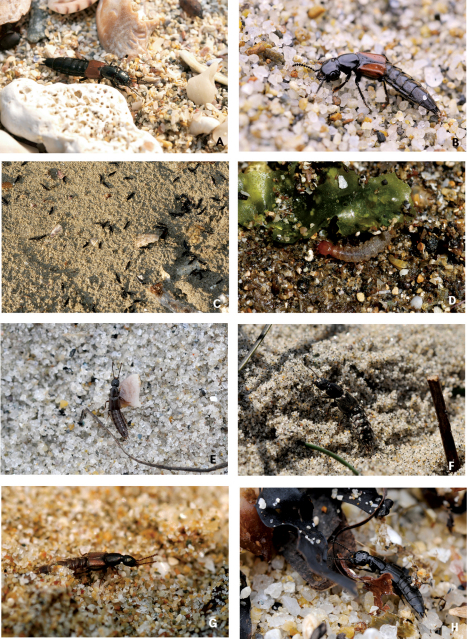
Coastal Staphylinidae. **A** *Phucobius simulator* on a sandy beach in Guryongpo, Korea **B** *Liusus hilleri* on a sandy beach in Donghae, Korea **C** Overwintering staphylinine species (*Cafius histrio*, *Liusus hilleri* and *Philonthus nudus*) under a wooden board on a sandy beach in Jindo Island, Korea **D** Larva of *Cafius* sp. under fresh seaweeds on a sandy beach in Jejudo Island, Korea **E** *Cafius bistriatus* on a sandy beach in North Carolina, USA **F** *Cafius seminites* under decaying seaweeds on a sandy beach in California, USA **G** *Cafius rufescens* on a sandy beach in Jindo Island, Korea **H** *Philonthus nudus* under decaying seaweeds on a sandy beach in Jindo Island, Korea.

Habitats of coastal staphylinids are drifted seaweed, the intertidal zone, sandy beaches, pebble beaches, rocky shores, muddy beaches and flats, salt marshes, and mangrove swamps (Figs 2–5), but these are not necessarily mutually exclusive. Staphylinidae are very often associated with plants and algae. These may be growing in estuaries (especially marshgrasses, such as *Spartina*), on rocky shores (green, brown, and red algae), or on sandy shores (diatoms). Or they may be plants or seagrasses (*Thalassia*, *Zostera*, etc.) torn loose and deposited on any of those shores. Few staphylinid studies have identified associated plants specifically or even generically. Often, the drifted seaweeds (i.e., seaweeds deposited onshore by the tide) are called ‘wrack’. Backlund (1945) used the term to include not only the brown alga *Fucus*, but many other algae, and the sea-grass *Zostera* (a vascular plant) deposited onshore. Barrett and Yonge (1958), in contrast, referred to large brown algae including the genus *Laminaria* as ‘kelp’, confining the term ‘wrack’ to moderately sized brown algae including *Pelvetia*, *Fucus*, and *Ascophyllum*. They apply these terms to growing seaweed as well as to seaweed deposited onshore. Moore and Legner (1973) defined wrack as drifted kelp. We employ the term ‘drifted seaweed’ to all algae deposited on shorelines, and we distinguish sea-grasses and marsh-grasses.

Drifted seaweed may be ephemeral and sparse and greatly subject to drying, to deep and decomposing and more or less permanent depending upon location and tides. Backlund (1945) published an ecological study of drifted seaweed in more or less permanent beds on seashores in Sweden and Finland, including its insect inhabitants. Staphylinids may occupy the sand under sparse accretions of seaweed. In thick seaweed beds, staphylinids inhabit the seaweed together with other invertebrates. Organisms that eat and decompose seaweed are more abundant, including amphipods (Crustacea) and seaweed flies (Diptera: Coelopidae), both fed upon by many predacious Staphylinidae, including *Cafius* spp. Amphipods (commonly called ‘beach-hoppers’ or ‘beach-fleas’) are innocuous with respect to humans, but coelopids, (i.e., *Coelopa* spp.) are more problematic. “The flies normally pass their whole lives on the weed [and] are abundant in the wrack all the year around [and] their numbers are not appreciated until something makes them come out into the open [and] they may fly in a band, a little above the ground [and] occasionally they stray far inland [and] can be a great nuisance when large numbers… congregate in shops, garages, and particularly dry-cleaners’ (Oldroyd 1965: 173–174).” By feeding on these flies, *Cafius* spp. and other staphylinids can be considered as beneficial species. Poleward movement of seaweed deposits and their coelopid inhabitants has been noted and attributed to global warming, and there is a suspicion that *Laminaria* is in decline and that *Fucus* is expected to decline in the future in the British Isles (Edward et al. 2007).

Rocky shores offer refuges for specialist staphylinids (those not found in other habitats) in crevices or empty barnacle shells that trap pockets of air. Such shorelines often support the growth of algae, and these algae exhibit zonation according to species (Jones 1968). Staphylinids likewise distribute themselves according to such zones where they find refuge among algal holdfasts (Jones 1968; Topp and Ring 1988b). Rocky shores not only provide a substrate for barnacles and living algae, with which some staphylinids are associated, but they also frequently include tidal pool habitats. Tidal pools are inhabited by a few staphylinids, e.g., *Rothium*. A few species such as *Micralymma marinum* (which is believed to prey on Collembola) attain higher elevations above sea level on rocky cliffs (Thayer 1985).

Coral reefs, even five km from the shoreline, are habitat for a species of *Brachypronomaea* (Sawada 1956). Its food there remained unknown until the abundant Collembola present at one such site suggested a probable food source (Ahn et al. 2003). Off the coast of the Aru Islands in the Indonesian Archipelago, among coral polyps, Fauvel (1878a) found unusual staphylinids and described the genera *Corallis* and *Polypea*. Their food and way of life have not yet been determined.

Shores may be graded from solid rock to boulders, cobbles, pebbles, gravels, and sand, and even finer particles typical of mud flats, salt marshes, and mangrove swamps. All of those substrates have their complement of coastal Staphylinidae.

Table 1 is our attempt to summarize information about habitats across all genera; it is incomplete because the available information is incomplete. We summarize existing information not just about habitats but also about behavior and physiology in the section on Habits, Habitats, and Classificatory Notes under the name of each genus.

**Table 1.** Genera of the Staphylinidae containing coastal species with their known numbers and habitats.

*Subfamily*	*Tribe*	*Genus*	*No. species*	*Tidal zone*	*Habitat*	*Microhabitat*
Microsilphinae		*Microsilpha*	1	not known	sand spit	not known
Omaliinae	Aphaenostemmini	*Giulianium*	3	HM	SB	UD, UP
	Omaliini	*Crymus*	2	not known	not known	US
		*Macralymma*	1	not known	SB	US
		*Micralymma*	2	ML	RH	RC
		*Omaliomimus*	10	ML	not known	US
		*Omalium*	4	not known	not known	US, UG, UD
Pselaphinae	Batrisini	*Arthromelus*	1	HM	MA	UD
		*Batriscenites*	2	HM	MA	UD
		*Batrisocenus*	1	HM	MA	UD
	Brachyglutini	*Brachygluta*	6	not known	SM	UG, UP
		*Briara*	1	PH	MA	UP, UD
		*Briaraxis*	1	not known	not known	UD, UP, US
		*Mangalobythus*	3	HM	MA	cavities in log
		*Nisaxis*	2	not known	SM	UD, UG
		*Pedisinops*	1	not known	coral reef	not known
		*Physoplectus*	4	not known	not known	UP
		*Prosthecarthron*	1	not known	MF, SM	UP
Aleocharinae	Aleocharini	*Aleochara*	16	PH, HM	SB, RH	US, UD
	Athetini	*Acticola*	1	not known	not known	US
		*Adota*	6	PH	SM, SB	US, UD
		*Atheta*	6	PH	SM, ES, SB	US, UD
		*Brundinia*	2	not known	SM, ES	UD
		*Halobrecta*	7	not known	SM, ES	US, UD
		*Hydrosmecta*	1	PH	SB	US
		*Iotarphia*	1	not known	not known	not known
		*Osakatheta*	1	HM	ES, MF	UP
		*Pontomalota*	2	PH, HM	SB	US, UD
		*Psammopora*	1	not known	not known	not known
		*Psammostiba*	5	PH, HM	SB	US
		*Tarphiota*	3	PH, HM	SB	US, UD
		*Thinusa*	2	PH, HM	SB	US, UD
	Diglottini	*Diglotta*	8	PH	SB, SM, ES, BS	UP, UB
	Falagriini	*Bryobiota*	2	PH, HM	SB	US, UD
		*Myrmecopora*	14	PH, HM	SB	US, UD
	Homalotini	*Cameronium*	5	HM	RH	RC, US
		*Heterota*	10	PH	SB	US, UD
		*Linoglossa*	1	not known	MA	not known
		*Paractocharis*	3	PH	SB	US
		*Pseudopasilia*	1	not known	BS	UP, US
		*Thinobiosus*	1	not known	SB	US
	Liparocephalini	*Amblopusa*	5	HM	BS	UP, UB, US
		*Baeostethus*	1	PH	SB	UP
		*Diaulota*	8	ML, VL	RH	EB, RC, RA
		*Halorhadinus*	3	HM	BS	UP, US
		*Ianmoorea*	1	PH	SB	UB
		*Liparocephalus*	4	ML, VL	RH	RC, RA
		*Paramblopusa*	2	HM	SB	UP
	Myllaenini	*Brachypronomaea*	4	ML	RH	coral reef
		*Bryothinusa*	30	HM, ML	SB, BS, MF, MA	UP, UB, RC, EB
		*Corallis*	1	VL	not known	under coral polyp
		*Lautaea*	1	not known	MA, MF	not known
		*Myllaena*	1	PH	BS	UP
		*Polypea*	1	VL	not known	under coral polyp
		*Rothium*	6	HM, ML	RH	RA
	Oxypodini	*Chilodera*	1	not known	not known	US
		*Dasydera*	1	not known	not known	US
		*Gyronotus*	1	not known	not known	not known
		*Oreuryalea*	1	PH	SB	US, UD
	Phytosini	*Actocharis*	2	PH	SB	UB
		*Arena*	2	PH	SB, MF	UB
		*Euphytosus*	1	not known	not known	not known
		*Phytosus*	8	not known	SB	UB, US, UD
	Incertae sedis	*Salinamexus*	3	PH	SB	US, UD, UP
Oxytelinae	Oxytelini	*Anotylus*	1	PH	SB	US, UD
		*Blediotrogus*	4	PH	SB	US
		*Pareiobledius*	3	PH	SB	US
		*Sartallus*	1	PH	SB	US, UD
	Thinobiini	*Bledius*	57	PH, HM	MF, SM, ES	UB, US, UD
		*Carpelimus*	1	PH	MF, SM, ES	UB, US, UD
		*Teropalpus*	9	PH	SB	US, UD
		*Thinobius*	3	PH, HM	BS, SB, SM, MF	US
Scydmaeninae	Cephenniini	*Cephennodes*	1	PH	BS	UP
Paederinae	Paederini	*Chetocephalus*	1	PH	SB	US
		*Medon*	4	PH	SB	US, UD
		*Ophioomma*	1	PH	SB	UD
		*Sunius*	2	PH	SB, MA	US
Staphylininae	Staphylinini	*Bisnius*	1	PH	SB	US
		*Cafius*	44	PH	SB	US, UG, UD
		*Gabronthus*	1	not known	not known	not known
		*Hadropinus*	1	PH	SB	US
		*Hadrotes*	2	PH	SB	US
		*Heterothops*	1	PH	SB	US, UD
		*Liusus*	2	PH	SB	US, UG, UD
		*Orthidus*	1	PH	SB	US,UP
		*Philonthus*	1	PH	SB, SM	US, UG, UD
		*Phucobius*	8	PH	SB	US, UG, UD
		*Quediocafus*	3	PH	SB	UG
		*Remus*	4	PH	SB	US, UG, UD
		*Thinocafius*	1	PH	SB	US
		*Thinopinus*	1	PH	SB	US

PH: proximal to high tide zone; HM: high to mid tide zone; ML: mid to low tide zone; VL: very low tide zone. BS: boulder shores (band of gravels/pebbles/cobbles); ES: estuary; MA: mangrove; MF: mud/sand flats; RH: rocky headland; SB: sandy beach; SM: salt marsh. EB: Inside of empty barnacles/shells; RA: rock with algae; RC: rock crevices; UB: under beach sand; UD: under debris; UG: under seagrasses; UP: under stones (gravels/pebbles/cobbles); US: under seaweeds.

### Zoogeography

Based on taxonomy and distribution, we provide some provisional ideas about the dispersion of the taxa.

## A Checklist of coastal Staphylinidae and their distribution

**MICROSILPHINAE**

*Microsilpha* Broun 1886: 889

*Microsilpha litorea* Broun 1886: 890 - SPO (NZ)

**OMALIINAE**

APHAENOSTEMMINI

*Giulianium* Moore 1976: 56 [rev Ahn and Ashe 1999]

*Giulianium alaskanum* Ahn and Ashe 1999: 162 - NPO (US: AK; JP: HK)

*Giulianium campbelli* Moore 1976: 57 - NPO (US: CA)

*Giulianium newtoni* Ahn and Ashe 1999: 163 - NPO (US: CA)

OMALIINI

*Crymus* Fauvel 1904b: 92

= *Arpediopsis* Cameron 1917a: 124

= *Arpediomimus* Cameron 1917f: 277

*Crymus antarticus* Fauvel 1904b: 93 - SAO (South Georgia; Falkland Islands)

= *falklandicus* (Cameron 1917a): 125

*Crymus kronii* (Kiesenwetter 1877): 161 SPO (NZ: Antipodes Island, Auckland Island, Campbell Island, South Island)

= *longiceps* (Broun 1914): 89

*Macralymma* Cameron 1945c: 179

*Macralymma punctiventre* Cameron 1945c: 179 - SPO (NZ)

*Micralymma* Westwood 1838: 129

*Micralymma marinum* (Strøm 1783): 65 - NAO (CA: NB, NL, NS, QC; US: MA, ME, NH; FR; GB: England, N. Ireland, Scotland; GL; IE; IS; NO), North and Baltic and Irish Seas (BE; DE; GB: England, Scotland, N. Ireland, Wales; NL; SE; RU: Karelia)

= *brevipenne* (Gyllenhal 1810): 234

= *johnstonis* Westwood 1838: 130

= *stimpsonii* LeConte 1863: 57

*Micralymma laticolle* Motschulsky 1860: 549 ACO (RU: Siberia) [probably does not belong to *Micralymma*]

*Omaliomimus* Jeannel 1940: 117

*Omaliomimus actobius* (Broun 1893): 1035 - SPO (NZ)

*Omaliomimus albipennis* (Kiesenwetter 1877): 162 - SPO (AU: Macquarie Island; NZ: Campbell Island, Auckland Island)

= *variipennis* (Lea 1920): 30

= *flavipennis* Cameron 1948: 723

*Omaliomimus carinigerus* (Broun 1893): 1036 - SPO (NZ)

*Omaliomimus chalmeri* (Broun 1893): 1037 - SPO (NZ)

*Omaliomimus conicus* (Fauvel 1878b): 484 - SPO (NZ)

*Omaliomimus laetipennis* (Broun 1910): 19 - SPO (NZ)

*Omaliomimus litoreus* (Broun 1886): 942 - SPO (NZ)

*Omaliomimus robustus* (Broun 1911): 96 - SPO (NZ: Chatham Islands, Pitt Island)

*Omaliomimus setipes* (Broun 1909b): 230 - SPO (NZ)

*Omaliomimus venator* (Broun 1909a): 98 SPO (AU: Macquarie Island; NZ: mainland, Antipodes Island, Auckland Island, Campbell Island, Snares Island)

*Omalium* Gravenhorst 1802: 111

*Omalium algarum* Casey 1885: 316 - NPO (CA: BC; US: CA, OR)

*Omalium laeviusculum* Gyllenhal 1827: 464 NAO (FR; GB: England, Scotland; IE; IS; NO), ACO (RU), North Sea (BE; DE; DK; GB: England, Scotland; NL), Irish Sea (GB: England, Wales; IE), Baltic Sea (FI; SE)

*Omalium riparium* Thomson 1857: 224 - NAO (ES; FR; GB: England, Scotland; MA; PT; DK: Faroes), Irish Sea (GB: England, Wales; IE), North Sea (BE; DE; DK; FR; GB: England, Scotland; NL), Baltic Sea (DE; DK; EE; FI; PL; SE), Mediterranean Sea (DZ; ES; FR; IT; MA; YU)

*Omalium rugulipenne* Rye 1864: 58 - NAO (GB: England, Scotland), Irish Sea (GB: England; IE), North Sea (BE; DE; FR; GB: England, Scotland; NL)

**PSELAPHINAE**

BATRISITAE

BATRISINI

*Arthromelus* Jeannel 1949: 149

*Arthromelus quadratus* Tanokuchi 1989: 88 - South China Sea (SG)

*Batriscenites* Jeannel 1952: 96

*Batriscenites celer* Tanokuchi 1989: 91 - South China Sea (SG)

*Batriscenites humicola* Tanokuchi 1989: 95 - South China Sea (SG)

*Batrisocenus* Raffray 1903: 48

*Batriscenites foveiterminalis* Tanokuchi 1989: 97 - South China Sea (SG)

GONIACERITAE

BRACHYGLUTINI

*Brachygluta* Thomson 1859: 54

*Brachygluta abdominalis* (Aubé 1833): 27 - NAO (eastern US; CA: NB, NS)

*Brachygluta cavicornis* (Brendel 1865a): 30 - NAO (eastern US: NY, MD, DC, VA)

*Brachygluta curvicera* (Motschulsky 1854): 4 - NAO (eastern US: NY)

*Brachygluta floridana* (Brendel 1865b): 257 - NAO (eastern US: NY, MD, VA, NC, SC, FL)

*Brachygluta luniger* (LeConte 1849): 87 - NAO (eastern US: MA, NY, NJ, MD, VA)

*Brachygluta ulkei* (Brendel 1866): 193 - NAO (eastern US: MD, DC, DE, VA, SC, GA, FL)

*Briara* Reitter 1882: 207

= *Gonatocerus* Schaufuss 1880: 30 [preoccupied]

= *Berlara* Reitter 1882: 206

*Briara bella* (Tanokuchi 1989): 101 - South China Sea (SG)

*Briaraxis* Brendel 1894: 158

*Briaraxis depressa* Brendel 1894: 159 - Caribbean Sea (US: FL; TT: Tobago, Costa Rica)

*Mangalobythus* Tanokuchi 1989: 104

*Mangalobythus acutifolius* Tanokuchi 1989: 109 - South China Sea (TH)

*Mangalobythus furcifer* Tanokuchi 1989: 106 - South China Sea (SG)

*Mangalobythus murphyi* Tanokuchi 1989: 111 - South China Sea (SG)

*Nisaxis* Casey 1886: 183

*Nisaxis maritima* Casey 1887: 468 - Gulf of Mexico (US: LA, MS, TX)

*Nisaxis tomentosa* (Aubé 1833): 33 - NAO (eastern US: CT, NY, NJ, DE, MD, DC, NC), Gulf of Mexico (FL, Al, MS, TX), Caribbean Sea

= *minuta* (Brendel 1865a): 30

= *cincinnata* Casey 1887: 466

*Pedisinops* Newton and Chandler 1989: 43

= *Pedinopsis* Raffray 1890: 102

= *Halohermatus* Sawada 1991: 148

*Pedisinops regulus* (Sawada 1991): 150 - NPO (JP: RY)

*Physoplectus* Reitter 1882: 210

= *Halorabyxis* Jeannel 1954: 338

= *Thalassomerus* Sawada 1992: 55

*Physoplectus irritans* Chandler 2001: 349 - SPO (AU: Queensland)

*Physoplectus miyakei* (Sawada 1992): 58 - NPO (JP: RY)

*Physoplectus reikoae* (Sawada 1992): 56 - NPO (JP: HN)

*Physoplectus vinsoni* (Jeannel 1954): 341 - INO (MU)

*Prosthecarthron* Raffray 1915: 2

*Prosthecarthron sauteri* Raffray 1915: 3 - NPO (KP; JP: HN, KY, SH, RY), SPO (TW; VN)

= *palpalis* (Löbl 1974): 97; 1977: 236

ALEOCHARINAE

ALEOCHARINI

*Aleochara* Gravenhorst 1802: 67 [rev Nearctic Klimaszewski 1984, southern Africa Klimaszewski and Jansen 1994, Palearctic Assing 1995]

*Aleochara (Coprochara) salsipotens* Bernhauer 1912c: 209 - SAO (NA; ZA), INO (ZA)

*Aleochara (Coprochara) squalithorax* Sharp 1888: 282 - East Sea (JP: HN; KR)

*Aleochara (Coprochara) sulcicollis* Mannerheim 1843: 225 NPO (CA: BC; US: AK, CA, OR, WA; MX: BN, BS), SPO (CL)

*Aleochara (Emplenota) albopila* (Mulsant and Rey 1852): 171 - Mediterranean Sea (FR; GR; IT; YU), Black Sea (BG), NAO (ES: Canary Islands)

*Aleochara (Emplenota) curtidens* Klimaszewski 1984: 101 - NPO (CA: BC; US: CA)

*Aleochara (Emplenota) fucicola* Sharp 1874: 9 - NPO, South China Sea, East Sea (CN: Hong Kong; JP: HN; KR)

= *variolosa* Weise 1877: 89

*Aleochara (Emplenota) litoralis* (Mäklin 1853): 182 - NAO (CA: NB, NL, NS, QC; US: FL, MA, NJ, NY, RI), NPO (CA: BC; US: AK, CA; MX: BN, BS, SO)

*Aleochara (Emplenota) obscurella* Gravenhorst 1806: 159 NAO (ES; FR; GB: England, Scotland; IE; NO), Irish Sea (GB: England, Wales; IE), North Sea (BE; DE; DK; GB: England, Scotland; NL; NO), Baltic Sea (DE; DK; PL; SE), Irish Sea (GB; IE)

= *algarum* Fauvel 1862b: 92

*Aleochara (Emplenota) pacifica* (Casey 1894): 290 NPO (CA: BC; US: CA, WA; MX: BN)

*Aleochara (Emplenota) phycophila* Allen 1937: 219 - NAO (GB)

*Aleochara (Emplenota) puetzi* (Assing 1995): 225 - East Sea (KR; RU: Sakhalin, Kamchatka)

*Aleochara (Polystomota) grisea* Kraatz 1856: 96 - NAO (FR; GB: England, Scotland; IE; MA; NO; PT), Irish Sea (GB: Wales), North Sea (BE; DE; DK; GB: England, Scotland; NL; NO), Baltic Sea (DE; DK; FI; PL; SE), Mediterranean Sea (DZ; ES; FR; IT; YU)

*Aleochara (Polystomota) punctatella* Motschulsky 1858b: 240 - NAO (England, Scotland; IE), Irish Sea (GB: England, Wales), North Sea (BE; GB: England, Scotland; NL), NAO (FR)

*Aleochara (Triochara) nubis* (Assing 1995): 232 - East Sea (RU: Sakhalin, Kamchatka)

*Aleochara (Triochara) trisulcata* Weise 1877: 88 - NPO, East Sea (CN: Hong Kong; JP: HN; KR)

*Aleochara (Triochara) zerchei* (Assing 1995): 231 - East Sea (KR; RU: Primorie, Sakhalin)

ATHETINI

*Acticola* Cameron 1944b: 618

*Acticola falkandica* Cameron 1944b: 619 - SAO (Falkland Islands)

*Adota* Casey 1910: 67 [rev Gusarov 2003a, b]

= *Panalota* Casey 1910: 71

= *Phyconoma* Easton 1971: 24

= *Halostiba* Yosii and Sawada 1976: 86

*Acticola colpophila* Gusarov 2003b: 16 - Gulf of California (MX: SO)

*Acticola gnypetoides* (Casey 1910): 69 - NPO (US: AK, CA)

*Acticola madida* (Bernhauer 1907): 400 - NPO (JP: HN, KY)

*Acticola magnipennis* (Bernhauer 1943): 184 - NPO, East Sea (JP: HN, KY; KR)

*Acticola maritima* (Mannerheim 1843): 224 - NPO (CA: BC; US: AK, CA), NAO (GB: England), North Sea (GB: England, Scotland)

= *massettensis* Casey 1910: 68

= *subintima* Casey1910: 68

= *setosetarsis* Casey 1910: 71

= *insons* Casey1911: 125

= *scolopacina* Casey 1911: 124

= *scortea* Casey 1911: 124

= *immigrans* (Easton 1971): 25 - North Sea (GB), SPO (NZ)

*Acticola ushio* (Sawada 1971a): 304 - NPO, East Sea (JP: HN, KY)

*Atheta* Thomson 1858: 36

*Atheta (Actophylla) varendorffiana* Bernhauer and Scheerpeltz [in Scheerpeltz 1934: 1637] - North Sea (DE)

= *varendorffi* Bernhauer 1908a: 334 [preoccupied]

*Atheta (Badura) ririkoae* Sawada 1989b: 285 - NPO, East Sea (JP: HN; KR)

*Atheta (Badura) tokiokai* (Sawada 1971a): 306 - NPO, East Sea (JP: HN, KY; KR)

*Atheta (Datomicra) acadiensis* Klimaszewski and Majka 2007: 49 NAO (CA: NB, NS, PE, QC)

*Atheta (Sipalatheta) algarum* Pace 1999b: 680 - South China Sea (CN: Hong Kong)

*Atheta novaescotiae* Klimaszewski and Majka [in Klimaszewski et al. 2006: 68] NAO (CA: NB, NS, NL, Sable Island; St Pierre and Miquelon: Miquelon)

*Brundinia* Tottenham 1949: 78

*Brundinia marina* (Mulsant and Rey 1853): 39 - NAO (FR; GB: England), North Sea (DE; GB: England, Scotland; NL), Baltic Sea (DE; SE), Irish Sea (GB: England, Wales; IE), Mediterranean Sea (FR; IT)

= *imbecilla* (G. Waterhouse) 1858: 6074 (and 1859: 16)

= *thinobia* Thomson 1861: 73

*Brundinia meridionalis* (Mulsant and Rey 1853): 38 - NAO (FR; GB: England), Irish Sea England, Wales), North Sea (BE; GB: England), Mediteranean Sea (FR; IT)

*Halobrecta* Thomson 1858: 35 [rev Gusarov 2004, mainly Nearctic]

*Halobrecta algae* (Hardy 1851): 78 - Baltic Sea (DK; EE; FI; RU: Karelia; SE), North Sea (BE; GB: England, Scotland), NAO (FR; NO), SPO (AU)

= *puncticeps* (Thomson 1852): 134

= *anthracina* Fairmaire 1852: 687 [synonymy based on statement by Fairmaire 1856: 424 but not otherwise verified]

*Halobrecta algophila* (Fenyes 1909): 419 - NPO (US: CA), SPO (NZ; CL: Palena), SAO (Tristan da Cunha: Inaccessible Island), NAO (GB), Mediterranean Sea (FR: Corsica)

= *barbarae* (Casey 1910): 18

= *importuna* (Casey 1911): 111

*Halobrecta cingulata* (Cameron 1920): 266 - South China Sea (SG)

= *consors* (Cameron 1920): 266

*Halobrecta discipula* Pace 1999a: 171 - SPO (AU; CL: Valparaíso)

*Halobrecta flavipes* Thomson 1861: 50 - NAO (NO), Baltic Sea (DK; EE; FI; SE), Mediterranean Sea (IT), North Sea (BE; DE; GB: England, Scotland), NAO (CA: NB; US: NY,VA), SPO (AU; CL: Llanquihue)

= *maritima* (G. Waterhouse) 1863: 137

= *halobrectha* (Sharp 1869): 139

= *pubes* (Mulsant and Rey 1873a): 660

= *puncticeps* sensu Mulsant and Rey 1875: 12

= *pocahontas* (Casey 1910): 19

= *vaticina* (Casey 1910): 19

= *incertula* (Casey 1910): 84

*Halobrecta halensis* Mulsant and Rey 1873b: 173 - Mediterranean Sea (FR)

*Halobrecta princeps* (Sharp 1869): 142 - NAO (GB: England)

*Hydrosmecta* Thomson 1858: 33

*Halobrecta subalgarum* Pace 1999b: 672 - South China Sea (CN: Hong Kong)

*Iotarphia* Cameron 1943: 352

*Iotarphia australis* Cameron 1943: 352 - Tasman Sea (AU: New South Wales)

*Osakatheta* Maruyama et al. 2008: 40

*Osakatheta yasukoae* Maruyama et al. 2008: 41 - NPO (JP: HN)

*Pontomalota* Casey 1885: 296 [rev Ahn and Ashe 1992]

*Pontomalota opaca* (LeConte 1863): 28 - NPO (CA: BC; US: CA, OR, WA; MX: BN)

= *californica* Casey 1885: 298

= *nigriceps* Casey 1885: 299

= *luctuosa* Casey 1911: 164

= *bakeri* Bernhauer 1912b: 170

*Pontomalota terminalia* Ahn and Ashe 1992: 356 - NPO (US: CA)

*Psammopora* Pace 2003: 154

*Pontomalota delittlei* Pace 2003: 157 - Tasman Sea (AU: Tasmania)

*Psammostiba* Yosii and Sawada 1976: 82 [rev Gusarov 2003b]

*Psammostiba comparabilis* (Mäklin 1853): 181 - NPO (CA: BC, US: AK, CA)

*Psammostiba hilleri* (Weise 1877): 90 - NPO, East Sea (JP: HN, KY)

= *multipunctata* (Sawada 1971a): 301

*Psammostiba jessoensis* (Brundin 1943): 22 - NPO (JP: HK, HN; RU: Maritime Territory)

*Psammostiba kamtschatica* (Brundin 1943): 21 - NPO (JP: HK; RU: Kamchatka, Kuril Islands)

*Psammostiba kenaii* Gusarov 2003b: 28 - NPO (CA: BC; US: AK, CA)

*Tarphiota* Casey 1894: 332 [rev Ahn 1996b, Ahn 1999, Klimaszewski et al. 2006]

*Tarphiota densa* (Moore 1978a): 115 - NPO (MX: BS, SO)

= *hirsutula* Casey 1910: 75

*Tarphiota fucicola* (Mäklin 1852): 306 - NPO (CA: BC), Gulf of California (MX: BC, SO)

= *debilicollis* Casey 1910: 75

= *pallidipes* Casey 1894: 333 - NPO (US: CA)

*Tarphiota geniculata* (Mäklin 1852): 308 - NPO (CA: BC; US: AK, CA, OR)

= *iota* Casey 1910: 76

= *insolita* Casey 1910: 76

= *litorina* Casey 1910: 75

= *seditiosa* Casey 1910: 76

*Thinusa* Casey 1894: 371 [rev Ahn 1997b]

*Thinusa fletcheri* Casey 1906: 353 - NPO (CA: BC; US: AK, CA, OR, WA)

= *divergens* Casey 1911: 213

= *nigra* Casey 1911: 214

= *robustula* Casey 1911: 215

*Thinusa maritima* (Casey 1885): 312 - NPO (CA: BC; US: CA, OR, WA; MX: BN)

= *obscura* Casey 1906: 354

DIGLOTTINI [key Pace 1986]

*Diglotta* Champion 1887: 228 (repeated 1899: 265) [rev Haghebaert 1991]

= *Diglossa* Haliday 1837: 252 [preoccupied]

*Diglotta brasiliensis* Caron and Ribeiro-Costa 2008: 53 - SAO (BR: Paraná)

*Diglotta legneri* Moore and Orth 1979a: 339 - NPO (US: CA)

*Diglotta littoralis* (Horn 1871): 331 - NAO (US: NJ)

*Diglotta maritima* Lea 1927: 277 - SPO (FJ: Levuka)

*Diglotta mersa* (Haliday 1837): 252 North Sea (BE; DE; DK; FR; GB: England; NL), Irish Sea (GB: N. Ireland, Wales), Mediterranean Sea (DZ; IT), NAO (CA: NB; GB: England, Scotland; IE)

= *Diglotta submarina* Fairmaire 1856: 468

*Diglotta pacifica* Fenyes 1921: 17 - NPO (US: CA, OR; MX: BN)

*Diglotta secqi* Pace 1992: 180 - Red Sea (DJ)

*Diglotta sinuaticollis* (Mulsant and Rey 1870): 176 NAO (GB: England), Irish Sea (GB: England, Wales; IE)

= *Diglotta crassa* (Mulsant and Rey 1870): 180

FALAGRIINI [rev Hoebeke 1985, phy Ahn and Ashe 1995]

*Bryobiota* Casey 1894: 367 [rev Ahn and Ashe 1995]

*Bryobiota bicolor* (Casey 1885): 311 - NPO (CA: BC; US: WA, OR, CA; MX: BN)

= *californica* (Scheerpeltz 1965): 49

*Bryobiota giulianii* (Moore 1978a): 113 - NPO (US: CA, WA)

*Myrmecopora* Saulcy 1864: 429 [rev Assing 1997a, b, Palaearctic]

*Myrmecopora (Lamproxenusa) algarum* (Sharp 1874): 12 - NPO, East Sea (JP: HN, Tokara Island)

= *miyamotoi* (Sawada 1955): 85

*Myrmecopora (Lamproxenusa) chinensis* Cameron 1944c: 158 South China Sea (CN: Hong Kong)

*Myrmecopora (Lamproxenusa) reticulata* Assing 1997b: 344 - NPO (RU: Far East; KP)

*Myrmecopora (Lamproxenusa) rufescens* (Sharp 1874): 11 - NPO, East Sea (JP: HN, KY)

*Myrmecopora (Paraxenusa) laesa* (Erichson 1839b): 73 Mediterranean Sea (IT; PT; FR; DZ; TN; HR; ES: Balearic Islands), NAO (ES: Canary Islands)

= *tenuicornis* (Küster 1854): no. 3

*Myrmecopora (Xenusa) anatolica* (Fagel 1969): 117 - Mediterranean Sea (CY; TR)

*Myrmecopora (Xenusa) bernhaueri* Koch 1936: 210 - Red Sea (EG; IL)

*Myrmecopora (Xenusa) boehmi* Bernhauer 1910: 259 Mediterranean Sea (FR: Corsica; IT; GR; TN; MA; CY)

= *sydowi* Bernhauer 1927b: 97

= *mediterranea* Fagel 1970: 152

*Myrmecopora (Xenusa) brevipes* Butler 1909: 29 NAO (FR; GB: England), Irish Sea (GB: Wales; IE), North Sea (GB: England) [but see Hammond 2000: 257]

= *oweni* Assing 1997a: 114 [*fide* Hammond 2000: 257]

*Myrmecopora (Xenusa) maritima* (Wollaston 1860): 51 NAO (ES: Canary Islands; PT: Madeira)

*Myrmecopora (Xenusa) minima* Bernhauer 1901a: 537 Black Sea (BG; RO) Mediterranean Sea (GR; MA)

= *buresi* Rambousek 1910: 19 [in Czech] and 21 [in French]

= *pamphylica* (Fagel 1969): 120

*Myrmecopora (Xenusa) simillima* (Wollaston 1864): 534 NAO (ES: Canary Islands; GB: England, Scotland; IE; NO), Baltic Sea (DK; DE), North Sea (GB: England, Scotland), Mediterranean Sea (FR; ES; PT; TN; DZ; EG), NAO (PT: Azores)

= *lohmanderi* Bernhauer 1927a: 167

*Myrmecopora (Xenusa) sulcata* (Kiesenwetter 1850): 218 Mediterranean Sea (AL; GR; FR: Corsica; IT: Sardinia, Sicily; HR), North Sea (GB), Black Sea (RO; BG; UA)

= *carica* Fagel1970: 155

*Myrmecopora (Xenusa) uvida* (Erichson 1840): 916 NAO (GB: England), Mediterranean Sea (AL; BG; CY; ES; GR; TN; IT; HR; YU), North Sea (BE; DE; GB: England; NL), NAO (FR), Black Sea (UA; GE)

= *meridiogallica* Scheerpeltz 1972: 101

HOMALOTINI

*Cameronium* Koch 1936: 202

*Cameronium flavipenne* Cameron 1944a: 318 - INO (SO; TZ: Zanzibar)

*Cameronium gomyi* Pace 1985: 622 - INO (Comoros)

*Cameronium lamuense* Pace 1994: 155 - INO (KE: Lamu)

*Cameronium obockianus* (Fauvel 1905): 146 - Red Sea [DJ; ET; YE: Barim (= Perim) Island]

*Cameronium sonorensis* Moore 1964a: 175 - Gulf of California (MX: SO)

*Heterota* Mulsant and Rey 1874: 194 [rev Park et al. 2008]

*Heterota arenaria* Cameron 1920: 251 - South China Sea (SG)

*Heterota brevicollis* (Bernhauer 1929): 187 - Red Sea [YE: Barim (= Perim) Island]

*Heterota gomyi* Jarrige 1973: 257 - INO (MG)

*Heterota obscura* Cameron 1938: 174 - INO (RE)

*Heterota pamphylica* Fagel 1969: 123 - Mediterranean Sea (TR)

*Heterota pictipennis* (Fauvel 1905): 142 - Red Sea (DJ; ET), INO (SO)

*Heterota plumbea* (G. Waterhouse) 1858: 6074 (and 1859: 15) - Mediterranean Sea (Europe), NAO (ES: Canary Islands; GB: England; US: FL), Irish Sea (GB: Wales), Caribbean Sea (JM; MX: QR)

= *fairmairii* (Brisout 1859): ccxviii

= *godelinaisei* (Fauvel 1862b): 92

= *trogophloeoides* (Wollaston 1864): 536

= *impressa* (Mulsant and Rey 1875): 459

*Heterota rougemonti* Pace 1993: 137 - Bali Sea (ID: Bali)

*Heterota sunjaei* Park and Ahn [in Park et al.] 2008: 111 - NPO (KR)

*Heterota vinsoni* Cameron 1947a: 118 - INO (MG; MU; RE, Comoros)

*Linoglossa* Kraatz 1859a: 10

*Linoglossa murphyi* Sawada 1991: 142 - South China Sea (SG)

*Paractocharis* Cameron 1917c: 154

*Paractocharis deharvengi* Pace 1990: 81 - Luzon Sea (PH: Mindoro)

*Paractocharis fucicola* Cameron 1917c: 155 - South China Sea (SG)

*Paractocharis orousseti* Pace 1990: 79 - Luzon Sea (PH: Mindoro)

*Pseudopasilia* Ganglbauer 1895: 211

*Pseudopasilia testacea* (Brisout 1863): 16 NAO (FR; GB: England), North Sea (BE), Mediterranean Sea (FR: mainland, Corsica; HR; IT; TN)

*Thinobiosus* Moore and Legner 1977: 468

*Thinobiosus salinus* Moore and Legner 1977: 469 - Gulf of California (MX: SO)

LIPAROCEPHALINI [phy Ahn and Ashe 1996b, Ahn et al. 2010]

*Amblopusa* Casey 1894: 355 [rev Ahn and Ashe 1996a, Zerche 1998]

= *Boreorhadinus* Sawada 1991: 147

*Amblopusa alaskana* Ahn and Ashe 1996a: 143 - NPO (US: AK)

*Amblopusa brevipes* Casey 1894: 356 - NPO (CA: BC; US: AK, CA)

= *pallida* Casey 1911: 212

*Amblopusa hokkaidona* Ahn and Ashe 1996a: 142 - NPO (JP: HK)

*Amblopusa magna* Zerche 1998: 106 - NPO (JP: HK; RU: Kuril Islands)

*Amblopusa pacifica* (Sawada 1991): 147 - NPO (JP: HK)

*Baeostethus* Broun 1909a: 96 [rev Leschen et al. 2002]

*Baeostethus chiltoni* Broun 1909a: 97 - SPO (NZ: Campbell, Auckland, Antipodes Island)

*Diaulota* Casey 1894: 354 [rev Ahn 1996a, Zerche 1998]

= *Genoplectes* Sawada 1955: 81

*Diaulota alaskana* Ahn 1996a: 278 - NPO (US: AK)

*Diaulota aokii* Sawada 1971b: 104 - NPO (JP: HK, HN; KR; US: AK)

*Diaulota densissima* Casey 1894: 354 - NPO (CA: BC; US: CA, OR, WA)

= *Diaulota insolita* Casey 1894: 355

*Diaulota fulviventris* Moore 1956a: 120 - NPO (US: CA; MX: BN)

*Diaulota harteri* Moore 1956a: 123 - NPO (US: CA; MX: BN)

= *Diaulota megacephala* Moore 1956a: 124

*Diaulota pacifica* Sawada 1971b: 101 - NPO (JP: HN; KR)

*Diaulota uenoi* (Sawada 1955): 82 - NPO (JP: HN, RY; KR)

*Diaulota vandykei* Moore 1956a: 125 - NPO (US: CA)

*Halorhadinus* Sawada 1971b: 92 [rev Ahn 2001]

*Halorhadinus aequalis* Sawada 1971b: 92 - NPO, East Sea (JP: HN; KR)

*Halorhadinus inaequalis* Sawada 1971b: 95 - NPO, East Sea (JP: HN; KR)

*Halorhadinus sawadai* Maruyama and Hayashi 2009: 72 - East Sea (JP: HN)

*Ianmoorea* Ahn 2006: 36

= *Moorea* Ahn 2004: 255

*Ianmoorea zealandica* (Ahn 2004): 258 - SPO (NZ: North Island, South Island)

*Liparocephalus* Mäklin 1853: 191 [rev Ahn 1997a, Maruyama and Ahn 2000b]

*Ianmoorea brevipennis* (Mäklin 1853): 192 - NPO (US: AK)

*Ianmoorea cordicollis* LeConte 1880: 177 - NPO (CA: BC; US: AK, CA, OR, WA)

*Ianmoorea litoralis* Kirschenblatt 1938: 532 - NPO (RU: Kuril Islands; JP: HK)

*Ianmoorea tokunagai* Sakaguti 1944: 20 - NPO (JP: SH, KY)

*Paramblopusa* Ahn and Ashe 1996a: 148 [rev Ahn and Ashe 1996a, Maruyama and Ahn 2000a]

*Paramblopusa borealis* (Casey 1906): 354 - NPO (CA: BC; US: AK, OR, WA; JP: HK)

*Paramblopusa eoa* Ahn and Maruyama 2000: 359 - NPO (RU: Kuril Islands)

MYLLAENINI [phy Ahn and Ashe 2004]

*Brachypronomaea* Sawada 1956: 197 [rev Ahn et al. 2003]

= *Thalassopora* Jarrige 1959: 63

*Brachypronomaea esakii* Sawada 1956: 197 - NPO (JP: RY)

*Brachypronomaea marchemarchadi* (Jarrige 1959): 64 - South China Sea (VN)

*Brachypronomaea nosybiana* (Jarrige 1959): 65 - INO (MG)

*Brachypronomaea sawadai* Jarrige 1964: 178 - SPO (NC)

*Bryothinusa* Casey 1904: 312 [rev Pace 1986, Haghebaert 1995, Ashe 2005]

= *Halesthenus* Sawada 1955: 83

*Bryothinusa algarum* Sawada 1971b: 90 - NPO (JP: HN, KY)

*Bryothinusa cameroni* (Fauvel 1904a): 74 - Red Sea [ER; YE: Kameran (= Cameran) Island, Barim (= Perim) Island]

= *Bryothinusa microphthalma* (Bernhauer 1929): 187

*Bryothinusa catalinae* Casey 1904: 313 - NPO (US: CA)

*Bryothinusa celebensis* (Fauvel 1878a): 301 - Celebes Sea (ID: Sulawesi)

*Bryothinusa chani* Moore and Legner 1971: 107 - South China Sea (CN: Hong Kong)

*Bryothinusa chengae* Ahn 1998: 335 - SPO (Caroline Island: Palau)

*Bryothinusa fluenta* Moore and Legner 1975: 111 - South China Sea (CN: Hong Kong)

*Bryothinusa gangjinensis* Ahn and Jeon 2004: 29 - NPO (KR)

*Bryothinusa grootaerti* Haghebaert 1995: 29 - Bismarck Sea (PG: Laing Island)

*Bryothinusa hauseri* Ashe 2005: 582 - South China Sea (MY: Malaya)

*Bryothinusa hongkongensis* Moore, Legner and Chan 1973: 77 South China Sea (CN: Hong Kong)

*Bryothinusa koreana* Ahn and Jeon 2004: 31 - NPO (KR)

*Bryothinusa madecassa* Pace 2008: 568 - INO (MG)

*Bryothinusa minuta* (Sawada 1955): 83 - NPO (JP: HN, RY; KR)

*Bryothinusa nakanei* (Sawada 1955): 85 - NPO (JP: RY; KR)

*Bryothinusa neoguineensis* Pace 2000a: 115 - Bismarck Sea (PG: Laing Island)

*Bryothinusa orousseti* Pace 1990: 66 - Luzon Sea (PH: Mindoro)

*Bryothinusa papuensis* Haghegbaert 1995: 31 - Bismarck Sea (PG: Cape Vogel Peninsula)

*Bryothinusa parvula* Haghebaert 1995: 27 - Bismarck Sea (PG: Laing Island)

*Bryothinusa perexilis* Pace 1994: 132 - INO (SO: Sar Uanle)

*Bryothinusa peyerimhoffi* (Fauvel 1904a): 73 Red Sea, Gulf of Akkaba, Mediterranean Sea (IL)

*Bryothinusa rothi* Moore and Legner 1975: 110 - Gulf of California (MX: SO)

*Bryothinusa sakishimana* Sawada 1991: 144 - NPO (JP: RY)

*Bryothinusa samoensis* Pace 1984: 67 - SPO (WS: Upolu Island)

*Bryothinusa sawadai* Moore, Legner and Chan 1973: 75 - South China Sea (CN: Hong Kong)

*Bryothinusa sinensis* Moore, Legner and Chan 1973: 76 - South China Sea (CN: Hong Kong)

*Bryothinusa subtilissima* (Cameron 1904): 157 Red Sea [YE: Barim (= Perim) Island; SO: Hartan Peninsula]

*Bryothinusa testacea* (Cameron 1904): 157 - Red Sea [YE: Barim (= Perim) Island]

*Bryothinusa testaceipennis* (Cameron 1919): 245 - South China Sea (SG)

*Bryothinusa tsutsuii* (Sawada 1955): 84 - NPO (JP: HN, RY)

= *BryothinusaB. serpentis* (Sawada 1955): 84

*Corallis* Fauvel 1878a: 212

*Corallis polyporum* Fauvel 1878a: 213 Arafura Sea [ID: Kepalauan Aru (Aru and Wokam), Kepalauan Kai (= Kei or Ke Island)]

*Lautaea* Sawada 1989a: 83

*Lautaea murphyi* Sawada 1989a: 85 - South China Sea (SG)

*Myllaena* Erichson 1837: 382

*Myllaena insipiens* Casey 1911: 237 - NAO, Gulf of Mexico (US: AL, FL, LA, NJ, PA)

*Polypea* Fauvel 1878a: 301

*Polypea coralli* Fauvel 1878a: 302 - Arafura Sea (ID: Kepalauan Aru)

*Rothium* Moore and Legner 1977: 460 [rev Ahn and Ashe 1996c]

*Rothium ashlocki* Ahn and Ashe 1996c: 247 - SPO (EC: Galapagos)

*Rothium evansi* Ahn and Ashe 1996c: 248 - SPO (EC: Esmeraldas, Guayas; PE: Piura)

*Rothium giulianii* Moore 1978b: 155 - NPO (MX: GU, SI)

*Rothium littoralis* Klimaszewski and Peck 1998: 228 - SPO (EC: Galapagos)

*Rothium pallidus* Ahn and Ashe 1996c: 247 - NPO (MX: GU)

*Rothium sonorensis* Moore and Legner 1977: 462 - Gulf of California (MX: SO)

OXYPODINI

*Chilodera* Cameron 1944b: 619

*Chilodera falklandica* Cameron 1944b: 620 - SAO (Falkland Islands)

*Dasydera* Cameron 1948: 731

= *Calonotus* Cameron 1945c: 171 [preoccupied]

= *Mecrona* Blackwelder 1952: 232

*Dasydera algophila* (Broun 1886): 941 - SPO (NZ: Mokohinau Island)

*Gyronotus* Cameron 1948: 731

= *Eurynotus* Cameron 1945c: 170 [preoccupied]

= *Marecon* Blackwelder 1952: 230

*Gyronotus rufipennis* (Broun 1880): 92 - SPO (NZ: North Island)

*Oreuryalea* Assing and Maruyama 2002: 210

*Oreuryalea watanabei* Assing and Maruyama 2002: 217 - NPO, East Sea (RU: Primorie, Sakhalin; JP: HK)

PHYTOSINI

*Actocharis* Sharp 1870: 279

*Actocharis readingii* Sharp 1870: 279 - NAO (FR; GB: England), Mediterranean Sea (DZ; FR: Corsica; HR; IT: mainland, Sardinia, Sicily; MT)

= *marina* Fauvel 1871: 159

*Actocharis cassandrensis* Assing 1992: 45 - Mediterranean Sea (GR)

*Arena* Fauvel 1862c: 292

*Arena fultoni* Cameron 1945c: 162 - SPO (NZ)

*Arena tabida* (Kiesenwetter 1850): 219 - NAO (FR; GB: England), North Sea (DE; DK; GB: England, Scotland; NL)

= *octavii* Fauvel 1862c: 292

*Euphytosus* Bernhauer and Scheerpeltz 1926: 552

[*Pseudophytosus* Haghebaert 1993: 161 is not a valid name]

= *Paraphytosus* Bernhauer 1922a: 236 [preoccupied]

*Euphytosus schenklingi* (Bernhauer 1922a): 236 - South China Sea (TW)

*Phytosus* Curtis 1838: 718

*Phytosus (Actosus) andalusiaensis* Haghebaert 1993: 161 Mediterranean Sea (ES: Andalusia)

*Phytosus (Actosus) balticus* Kraatz 1859b: 52 - NAO (ES: Canary Islands; ES; FR; GB: England; IE; NO; PT), Irish Sea (GB: England), North Sea (BE; DE; GB: England, Scotland; NL), Baltic Sea (DE; DK; SE), Mediterranean Sea (DZ; FR; IT; MA; TN)

*Phytosus (Actosus) holtzi* Bernhauer 1935: 48 - Mediterranean Sea (GR: Crete)

*Phytosus (Actosus) nigriventris* (Chevrolat 1843): 42 - NAO (ES; FR; GB: England; PT), Irish Sea (GB: England), North Sea (BE; NL), Mediterranean Sea (IT; MA)

= *minyops* Wollaston 1864: 531

*Phytosus (Actosus) schatzmayri* Bernhauer 1941: 95 - NAO (PT: Azores)

*Phytosus* (*s. str.*) *caribeanus* Haghebaert 1993: 163 - Caribbean Sea (GP)

*Phytosus* (*s. str.*) *fenyesi* (Bernhauer 1915a): 315 - NAO (SN)

= *senegalensis* Wendeler 1930: 252

*Phytosus* (*s. str.*) *spinifer* Curtis 1838: 718 - NAO (ES: Canary Islands; FR; GB: England; IE; MA; PT), Mediterranean Sea (DZ; EG; ES; FR; GR; IT; MA; TN), Black Sea (BG; RO; TR), Baltic Sea (DE; DK; FI; SE), North Sea (BE; DE; DK; GB: England; NL)

= *dimidiatus* Wollaston 1865: 453

Incertae sedis

*Salinamexus* Moore and Legner 1977: 463 [rev Jeon and Ahn 2007]

= *Biophytosus* Moore and Legner 1977: 465

*Salinamexus browni* Moore and Legner 1977: 464 - Gulf of California (MX: SO)

*Salinamexus koreanus* Jeon and Ahn 2007: 193 - NPO (KR)

*Salinamexus reticulatus* (Moore and Legner 1977): 466 - Gulf of California (MX: SO)

OXYPODINI

*Chilodera* Cameron 1944b: 619

*Chilodera falklandica* Cameron 1944b: 620 - SAO (Falkland Islands)

*Dasydera* Cameron 1948: 731

= *Calonotus* Cameron 1945c: 171 [preoccupied]

= *Mecrona* Blackwelder 1952: 232

*Dasydera algophila* (Broun 1886): 941 - SPO (NZ: Mokohinau Island)

*Gyronotus* Cameron 1948: 731

= *Eurynotus* Cameron 1945c: 170 [preoccupied]

= *Marecon* Blackwelder 1952: 230

*Gyronotus rufipennis* (Broun 1880): 92 - SPO (NZ: North Island)

*Oreuryalea* Assing and Maruyama 2002: 210

*Oreuryalea watanabei* Assing and Maruyama 2002: 217 - NPO, East Sea (RU: Primorie, Sakhalin; JP: HK)

PHYTOSINI

*Actocharis* Sharp 1870: 279

*Actocharis readingii* Sharp 1870: 279 - NAO (FR; GB: England), Mediterranean Sea (DZ; FR: Corsica; HR; IT: mainland, Sardinia, Sicily; MT)

= *marina* Fauvel 1871: 159

*Actocharis cassandrensis* Assing 1992: 45 - Mediterranean Sea (GR)

*Arena* Fauvel 1862c: 292

*Arena fultoni* Cameron 1945c: 162 - SPO (NZ)

*Arena tabida* (Kiesenwetter 1850): 219 - NAO (FR; GB: England), North Sea (DE; DK; GB: England, Scotland; NL)

= *octavii* Fauvel 1862c: 292

*Euphytosus* Bernhauer and Scheerpeltz 1926: 552

[*Pseudophytosus* Haghebaert 1993: 161 is not a valid name]

= *Paraphytosus* Bernhauer 1922a: 236 [preoccupied]

*Euphytosus schenklingi* (Bernhauer 1922a): 236 - South China Sea (TW)

*Phytosus* Curtis 1838: 718

*Phytosus (Actosus) andalusiaensis* Haghebaert 1993: 161 Mediterranean Sea (ES: Andalusia)

*Phytosus (Actosus) balticus* Kraatz 1859b: 52 - NAO (ES: Canary Islands; ES; FR; GB: England; IE; NO; PT), Irish Sea (GB: England), North Sea (BE; DE; GB: England, Scotland; NL), Baltic Sea (DE; DK; SE), Mediterranean Sea (DZ; FR; IT; MA; TN)

*Phytosus (Actosus) holtzi* Bernhauer 1935: 48 - Mediterranean Sea (GR: Crete)

*Phytosus (Actosus) nigriventris* (Chevrolat 1843): 42 - NAO (ES; FR; GB: England; PT), Irish Sea (GB: England), North Sea (BE; NL), Mediterranean Sea (IT; MA)

= *minyops* Wollaston 1864: 531

*Phytosus (Actosus) schatzmayri* Bernhauer 1941: 95 - NAO (PT: Azores)

*Phytosus* (*s. str.*) *caribeanus* Haghebaert 1993: 163 - Caribbean Sea (GP)

*Phytosus* (*s. str.*) *fenyesi* (Bernhauer 1915a): 315 - NAO (SN)

= *senegalensis* Wendeler 1930: 252

*Phytosus* (*s. str.*) *spinifer* Curtis 1838: 718 - NAO (ES: Canary Islands; FR; GB: England; IE; MA; PT), Mediterranean Sea (DZ; EG; ES; FR; GR; IT; MA; TN), Black Sea (BG; RO; TR), Baltic Sea (DE; DK; FI; SE), North Sea (BE; DE; DK; GB: England; NL)

= *dimidiatus* Wollaston 1865: 453

Incertae sedis

*Salinamexus* Moore and Legner 1977: 463 [rev Jeon and Ahn 2007]

= *Biophytosus* Moore and Legner 1977: 465

*Salinamexus browni* Moore and Legner 1977: 464 - Gulf of California (MX: SO)

*Salinamexus koreanus* Jeon and Ahn 2007: 193 - NPO (KR)

*Salinamexus reticulatus* (Moore and Legner 1977): 466 - Gulf of California (MX: SO)

**OXYTELINAE**

OXYTELINI

*Anotylus* Thomson 1859: 44

*Anotylus maritimus* Thomson 1861: 131 - NAO (FR; GB: England, Scotland; NO),Baltic Sea (SE), North Sea (BE; DK; GB: England; NL), Mediterranean Sea (IT: Sicily; TN)

= *perrisii* Fauvel 1862a: xxiv

= *oceanus* Fauvel 1862c: 292

*Blediotrogus* Sharp 1900: 234

*Blediotrogus cordicollis* (Broun 1907): 57 - SPO (NZ: Chatham Islands)

*Blediotrogus cribricollis* Fauvel 1900: 184 - SPO (NZ)

*Blediotrogus fauveli* (Bernhauer and Schubert 1911): 129 - SPO (AU)

*Blediotrogus guttiger* Sharp 1900: 234 - SPO (NZ)

*Pareiobledius* Bernhauer 1934: 495

*Pareiobledius alutellus* (Bernhauer 1934): 495 - SAO (ZA)

*Pareiobledius madegassa* Scheerpeltz 1969: 127 - INO (MG)

*Pareiobledius pruinosus* (Bernhauer 1912a): 178 - SAO (ZA)

*Sartallus* Sharp 1871b: 217

*Sartallus signatus* Sharp 1871b: 217 - SPO (AU: South Australia)

THINOBIINI

*Bledius* Leach 1819: 174

aequatorialis gp

*Bledius aequatorialis* Mutchler 1925: 225 - SPO (EC: mainland, Galapagos Islands)

*Bledius ceratus* Blackwelder 1943: 118 - Caribbean Sea (BS; CU; DO; HT; JM; US: FL)

*Bledius susae* Herman 1983: 98 - Gulf of Mexico (US: TX, South Padre Island)

armatus gp

*Bledius fenyesi* Bernhauer and Schubert 1911: 129 - NPO (US: CA; MX: BN, BS)

= *lecontei* Bernhauer 1905: 14 [preoccupied]

*Bledius monstratus* Casey 1889b: 46 - NPO (CA: BC; US: OR, CA)

basalis gp

*Bledius cordatus* (Say 1834): 461 - NAO and Gulf of Mexico (US: NY-FL, MS, TX)

*Bledius doderoi* Bondroit 1912: 66 - Mediterranean Sea (GR; IT)

*Bledius fergussoni* Joy 1912: 44 NAO (GB: England, N. Ireland, Scotland; IE), Baltic Sea (DE; EE; FI; PL; SE; RU: Karelia), North Sea (BE; FR; GB: England, Scotland; NO), Irish Sea (GB: England, Wales), Black Sea (RO), Mediterranean Sea (FR; MA; TN)

*Bledius gradensis* Bernhauer 1929: 183 - Mediterranean Sea (IT)

*Bledius minor* Mulsant and Rey 1878: 634 - Mediterranean Sea (AL; FR; IT)

*Bledius neglectus* Casey 1889b: 69 - NAO (CA: NL, NB, NS; US: ME-GA)

*Bledius subniger* Schneider 1898: 62 NAO (GB: England, Scotland; IE), Irish Sea (GB: England, Wales, IE), North Sea (DE; GB: England, Scotland; NL), Mediterranean Sea (ES; TN)

*Bledius thinopus* Herman 1976: 86 - Gulf of Mexico (US: FL, AL, TX)

*Bledius turbulentus* Casey 1889b: 70 - Gulf of Mexico (MX: QR, YU; US: FL, MS)

bonariensis gp

*Bledius bonariensis* Bernhauer 1912a: 168 - SAO (AR; BR; UY)

forcipatus gp

*Bledius actitus* (Herman 1972): 127 - Gulf of Mexico (US: TX)

*Bledius litoreus* (Herman 1972): 129 - Gulf of Mexico (US: FL)

fratellus gp

*Bledius fratellus* Eppelsheim 1885: 144 - SAO (GH; NG; SN)

furcatus gp

*Bledius maritimus* Bernhauer 1923: 176 - Red Sea (SU)

gigantulus gp

*Bledius marinus* Bernhauer 1922b: 168 - INO (SC: Aldabra Islands)

*Bledius philippinus* Bernhauer 1912d: 248 - South China Sea (PH: Luzon)

*Bledius yezoensis* Nakane 1963: 21 - NPO (JP; KR)

infans gp

*Bledius helferi* Fauvel 1904b: 112 - INO (IN; MM)

*Bledius infans* Rottenberg 1870: 36 - Mediterranean Sea (DZ; IT; LY; TN), Red Sea (YE)

*Bledius renominatus* Cameron 1914: 203 - INO (ET; SO)

= *bernhaueri* Cameron 1912: 28 [preoccupied]

lamelliceps gp

*Bledius hasticeps* Bernhauer 1937: 583 - INO (MG; TZ)

pulchellus gp

*Bledius pulchellus* Kraatz 1859a: 169 - INO (IN; LK; Chagos Archipelago)

punctatissimus gp

*Bledius albomarginatus* Bernhauer 1922a: 225 - South China Sea (TW)

*Bledius amplicollis* Fauvel 1900: 185 - SPO (NZ)

*Bledius bidentifrons* Broun 1912: 401 - SPO (NZ)

*Bledius buehleri* Scheerpeltz 1957b: 226 - Timor Sea (ID: Sumba)

*Bledius buettikeri* Coiffait 1981a: 241 - Red Sea (SA)

*Bledius capensis* Cameron 1945a: 708 - SAO (ZA)

*Bledius caribbeanus* Blackwelder 1943: 113 - Caribbean Sea (JM; DO-TT)

*Bledius caroli* Blackburn 1888: 13 - SPO (AU)

*Bledius exiguus* Scheerpeltz 1933: 1114 - SPO (AU)

= *minor* Bernhauer 1920: 6 [preoccupied]

*Bledius fernandezi* Bernhauer 1939: 234 - SAO (UY)

*Bledius fossiventris* Fauvel 1889: 252 - SPO (NC)

*Bledius injucundus* Blackburn 1888: 14 - SPO (AU)

*Bledius maindroni* Fauvel 1903: 151 - INO (IN)

*Bledius michaelseni* Bernhauer 1915b: 313 - SAO (NA)

*Bledius microcephalus* Fauvel 1901: 72 - NPO (CO), Caribbean Sea (TT: Trinidad)

*Bledius orientalis* Bernhauer and Schubert 1911: 133 - Red Sea (DJ)

= *lividipes* Fairmaire 1892: 90 [preoccupied]

*Bledius pontilis* Blackburn 1902: 22 - SPO (AU)

*Bledius punctatissimus* LeConte 1877: 226 NAO (US: MDFL), Gulf of Mexico (US; MX: VC), Caribbean Sea (JM; PR), Gulf of California (MX), NPO (CO; EC)

*Bledius salinus* Cameron 1947b: 704 - SPO (NZ)

*Bledius tristis* Aubé 1843: 92 Mediterranean Sea (AL; DZ; ES; FR; IT; TN), Red Sea (SN)

rugosicollis gp

*Bledius bituberculatus* Cameron 1940: 183 - Andaman Sea (MY)

verres gp

*Bledius albopubescens* Cameron 1941: 434 - South China Sea (PH: Luzon)

*Bledius arenicola* Fauvel 1904b: 112 - Arabian Sea [IN: Malabar, Mahé (= Mayyazhi)]

*Bledius fraterculus* Cameron 1936: 40 - Andaman Sea (MY)

*Bledius jacobsoni* Cameron 1928: 106 - INO (ID: Sumatra)

*Bledius madagascariensis* Bernhauer 1901b: 169 - INO (MG)

*Bledius marginalis* Cameron 1945a: 707 - SAO (ZA: Cape Province)

*Bledius parens* Cameron 1941: 434 - South China Sea (PH: Luzon)

*Bledius perrieri* Fauvel 1904c: 305 - INO (MG)

*Bledius petzi* Bernhauer 1908b: 104 - INO (TZ)

*Carpelimus* Leach 1819: 174

*Carpelimus lucidus* (Cameron 1944a): 312 - INO (TZ: Zanzíbar)

*Teropalpus* Solier 1849: 330

= *Trogolinus* Sharp 1900: 231

*Teropalpus coloratus* (Sharp 1900): 231 - SPO (NZ)

*Teropalpus lithocharinus* (LeConte 1877): 245 - NPO (US: CA, WA)

*Teropalpus luniger* (Fauvel 1868): 40 - SPO (CL)

*Teropalpus maritimus* (Broun 1903): 615 - SPO (NZ)

*Teropalpus pictipes* (Lea 1910): 126 - SPO (AU: Tasmania)

*Teropalpus senex* (Fauvel 1868): 40 - SPO (CL)

*Teropalpus skottsbergii* (Bernhauer 1921): 41 - SPO (CL: Juan Fernandez Island)

*Teropalpus suturalis* Solier 1849: 331 - SPO (CL)

*Teropalpus unicolor* (Sharp 1900): 232 - SPO (AU, NZ), INO (ZA), NAO (GB: England)

= *anglicanus* (Sharp 1900): 232

*Thinobius* Kiesenwetter 1844: 355

= *Yosiityphlus* Sawada 1971c: 327

*Thinobius frizzelli* Hatch 1957: 94 - NPO (CA: BC; US: CA, WA; MX: BN)

*Thinobius marinus* Cameron 1917d: 155 - South China Sea (SG)

*Thinobius kuroshio* (Sawada 1971c): 327 - NPO (JP: HN)

**SCYDMAENINAE**

CEPHENNIINI

*Cephennodes* Reitter 1884: 420

= *Chelonoidum* Strand 1935: 285

*Cephennodes araiorum*(Jałoszyński 2003): 226 - NPO (JP: HN)

**PAEDERINAE**

PAEDERINI

*Chetocephalus* Cameron 1944a: 314

*Chetocephalus maritimus* Cameron 1944a: 314 - INO (MU)

*Medon* Stephens 1833: 273

*Medon marinus* Cameron 1944a: 313 - INO (MU)

*Medon pocoferus* (Peyron 1857): 718 - Mediterranean Sea (DZ; FR; IT; TN), NAO (FR; GB: England)

= *maritimus* Aubé 1863: 36

*Medon prolixus* (Sharp 1874): 65 - NPO, East Sea (JP: HN)

*Medon rubeculus* Sharp 1889: 264 - NPO (JP: HN), South China Sea (CN: Hong Kong)

*Ophioomma* Notman 1920: 704

*Ophioomma rufa* Notman 1920: 705 - Gulf of Mexico (US: FL)

*Sunius* Stephens 1829: 24

*Sunius ferrugineus* (Bierig 1934b): 326 - Caribbean Sea (CU; JM)

*Sunius minutus* (Casey 1905): 180 - NAO (US: FL)

**STAPHYLININAE**

STAPHYLININI

PHILONTHINA

*Bisnius* Stephens 1829: 23

*Bisnius macies* (Sharp 1874): 41 - NPO (JP; KR)

*Cafius* Stephens 1829: 23

*Cafius aguayoi* Bierig 1934a: 66 - NAO (US: CT, MA)

*Cafius algarum* (Sharp 1874): 38 - NPO, East Sea (JP: HN; KR), South China Sea (CN: Hong Kong)

*Cafius algophilus* Broun 1894: 419 - SPO (NZ)

*Cafius andamanensis* Coiffait 1981b: 337 - INO (Andaman Island)

*Cafius australis* (Redtenbacher 1867): 28 - SPO (AU: New South Wales, Victoria)

= *areolatus* Fauvel 1877: 251

*Cafius bistriatus* (Erichson 1840): 502

= *bilineatus* (Erichson 1840): 503

ssp. *bistriatus* (Erichson 1840): 503 - NAO (CA: NB, NL, NS, QC; US: MA, MD, ME, NJ, NY, RI, VA, FL; BM, BS), Gulf of Mexico (MX: CA, VC; US: FL, TX), Caribbean Sea (AG; BB; CU; DO; GD; GP; JM; KN; LC; MS; PR; TT; VE; VI; MX: QR)

ssp. *fulgens* Frank [in Frank et al. 1986]: 153 - NPO (US: CA; MX: BS), Gulf of California (MX: BN, BS, SO)

*Cafius bisulcatus* (Solier 1849): 314 - SPO (CL)

*Cafius bryanti* Cameron 1943: 343 - SPO (AU)

*Cafius canescens* (Mäklin 1852): 313 - NPO (US)

*Cafius caribeanus* Bierig 1934a: 68 - Caribbean Sea (AG; CU; DM; GD; GP; JM; PR; VI; VE; US: FL)

*Cafius catenatus* Fauvel 1877: 256 - SPO (AU: New South Wales)

= *velutinus* Fauvel 1877: 256

*Cafius caviceps* Broun 1886: 942- SPO (NZ)

= *puncticeps* White 1846: 6

*Cafius ceylonicus* Bernhauer 1902: 29 - INO (LK)

*Cafius cicatricosus* (Erichson 1840): 454 Mediterranean Sea (southern Europe; IT), NAO (FR; GB: England)

= *sculticeps* (Motschulsky 1858a): 649

*Cafius decipiens* (LeConte 1863): 40 - NPO (US; MX)

*Cafius flicki* Vauloger 1897: 238 - Mediterraean Sea (IT; LY; TN)

*Cafius fonticola* (Erichson 1840): 501 - Red Sea (EG), INO (SO)

*Cafius fucicola* Curtis 1830: pl. 323 - NAO (GB: England; IE), Irish Sea (GB: N. Ireland, Wales; IE), North Sea (FR; GB)

*Cafius gigas* Lea 1929: 204 - SPO (AU: Lord Howe Island)

*Cafius histrio* (Sharp 1874): 37 - NPO, East Sea (CN: Hong Kong; JP: HN; KP; KR)

*Cafius lithocharinus* (LeConte 1863): 38 - NPO (CA; US; MX)

*Cafius litoreus* (Broun 1880): 108 - SPO (NZ)

*Cafius luteipennis* Horn 1884: 237 - NPO (CA; US; MX)

*Cafius maritimus* (Broun 1880): 109 - SPO (NZ)

*Cafius martini* Cameron 1927: 251 - Red Sea (SA; YE)

= *arrowi* Bernhauer 1931b: 234

*Cafius mimulus* (Sharp 1874): 38 - NPO, East Sea (JP: HN; KR)

*Cafius mutatus* Gemminger and Harold 1868: 590 - NPO (US; CA)

= *femoralis* (Mäklin 1853): 189

*Cafius nasutus* Fauvel 1877: 257 [and 1879: 84] - SPO (FJ)

*Cafius nauticus* (Fairmaire 1849): 288 - NPO (CN; JP; TW; US: HI), SPO (AU; FR: Tahiti; NC), INO (SO; LK; MU), Red Sea [YE: Barim (= Perim) Island]

= *longipennis* (Walker 1858): 205

= *puncticollis* (Boheman 1858): 31

= *parallelus* (Kraatz 1859a): 99

= *densiventris* Fauvel 1877: 258

*Cafius opacus* (LeConte 1863): 40 - NPO (western US; CA; MX)

= *dubius* (LeConte 1863): 39

*Cafius pacificus* (Erichson 1840): 501 - SPO (AU: New South Wales, Queensland, Tasmania, Victoria)

= *littoralis* Fauvel 1877: 254

*Cafius quadriimpressus* (White 1846): 6 - SPO (NZ)

= *expuncticollis* Koch 1936: 180

*Cafius ragazzii* Gestro 1889: 32 - Red Sea, INO (SO)

*Cafius rufescens* Sharp 1889: 44 - NPO, East Sea (JP: HN; KR), South China Sea (CN: Hong Kong)

*Cafius rufifrons* Bierig 1934a: 68 - Caribbean Sea (CU), NAO (US: FL)

*Cafius sabulosus* Fauvel 1877: 253 - SPO (AU: New South Wales, Queensland)

= *postseriatulus* Koch 1936: 179

*Cafius seminitens* Horn 1884: 236 - NPO (CA; US; MX)

*Cafius seriatus* Fauvel 1877: 255 - SPO (AU)

*Cafius subtilis* Cameron 1922: 121 - Caribbean Sea (AG; CU; DM; GP; JM; KN; MS; PR; TT; VI), Gulf of Mexico (US: FL), NAO (US: FL; BM)

*Cafius sulcicollis* (LeConte 1863): 40 - NPO (US; MX)

*Cafius velutinus* Fauvel 1877: 256 - SPO (AU)

*Cafius vestitus* (Sharp 1874): 37 - NPO, East Sea (JP: HN; KP; KR)

*Cafius xantholoma* (Gravenhorst 1806): 41 - NAO (ES: Canary Islands; FR; GB: England, Scotland; IE; IS; PT), Irish Sea (GB: England, Wales, N. Ireland; IE), North Sea (BE; DE; FR; GB: England, Scotland; NL; NO), Baltic Sea (DE; DK; EE; FI; PL; RU: Karelia; SE), Mediterranean Sea (DZ; EG; ES; FR; GR; IT; MA; TN; TR), Black Sea (UA)

= *lateralis* Stephens 1833: 246

= *littoralis* Stephens 1833: 247

= *tessellatus* Stephens 1833: 247

= *variegatus* (Erichson 1840): 453

= *variolosus* (Sharp 1871a): 181

= *keysianus* Donisthorpe 1930: 97

= *heroopoliticus* Koch 1936: 169

*Cafius zealandicus* Cameron 1947b: 705 - SPO (NZ)

*Gabronthus* Tottenham 1955: 178

*Gabronthus maritimus* (Motschulsky 1858a): 661 - NAO (ES: Canary Islands), Mediterranean Sea (CY; DZ; EG; FR; GR; IL; IT; LB; LY; MA; TR), Red Sea (DJ; ET; SA), INO (MU; RE), South China Sea (ID; MY; SG; VN), NPO (JP; TW)

= *mimulus* (Rottenberg 1870): 30

*Orthidus* Mulsant and Rey 1876: 339

*Orthidus cribratus* (Erichson 1840): 431 - Mediterranean Sea, NAO

ssp. *cribratus* (Erichson 1840): 431 - Mediterranean Sea (IT)

ssp. *atlanticus* Coiffait 1956: 221 - NAO (ES; FR; PT; MA)

*Philonthus* Stephens 1829: 23

*Philonthus nudus* Sharp 1874: 36 - NPO (CA: BC; US: WA; RU: Kuril Islands), NPO, East Sea (KR; JP: HK, HN, KY)

*Phucobius* Sharp 1874: 35

*Phucobius africanus* Bernhauer 1937: 617 - INO (TZ: western Usambara)

*Phucobius congruus* (Walker 1858): 205 - INO (LK)

= *punctilinea* (Walker 1858): 205

= *horni* (Bernhauer 1902): 28

*Phucobius cupreipennis* Cameron 1918: 89 - Java Sea (MY; SG)

*Phucobius densipennis* Bernhauer 1931a: 131 - NPO (JP: RY)

*Phucobius pectoralis* (Boheman 1858): 31 - ?sea (CN: province not stated)

*Phucobius semiaereus* Cameron 1934: 22 - SPO (New Hebrides)

*Phucobius simulator* Sharp 1874: 35 - NPO, East Sea (KR; JP: HN, KY; RU: Primorie)

*Phucobius tricolor* Bernhauer 1917: 125 - South China Sea (CN: Hong Kong; TW)

*Remus* Holme 1837: 64

*Remus corallicola* (Fairmaire 1849): 289 SPO (AU; FJ; NC), INO (LK; MG; MU; SC; SO), Red Sea (DJ; YE), South China Sea (CN: Hong Kong), Java Sea (ID; MY; SG)

= *occidentalis* Blackburn 1888: 48

*Remus filum* Kiesenwetter 1849: 19 Mediterranean Sea (IT; LY; YU), Black Sea, NAO (FR; DE; BG; RO; TR; EG; LY; HR), INO (SO)

*Remus pruinosus* (Erichson 1840): 510 - NAO (ES: Canary Islands; PT; NL; FR; RU), North Sea (BE; FR; NL), Mediterranean Sea (IT; TR)

*Remus sericeus* (Holme 1837): 64 - NAO (ES: Canary Islands; FR; GB: England; PT: Madeira; NO), Baltic Sea (DE; DK; SE), North Sea (DE; DK; GB: England; NL), Irish Sea (GB: Wales), Mediterranean Sea (EG; ES; FR; GR; IT; LY; TR), Black Sea (BG), INO (MU), SPO (AU: South Australia, Tasmania, Victoria, Western Australia)

= *aegyptiacus* Motschulsky 1858a: 665

= *obscuricornis* Koch 1936: 170

*Thinocafius* Steel 1949: 309

*Thinocafius insularis* Steel 1949: 309 - SPO (NZ: Chatham Islands)

QUEDIINA

*Heterothops* Stephens 1829: 23

*Heterothops asperatus* Smetana 1971: 34 - NPO (CA: BC; US: CA)

*Quediocafus* Cameron 1945b: 791

*Quediocafus hudsoni* Cameron 1945b: 791 - SPO (NZ)

*Quediocafus insolitus* (Sharp 1886): 379 - SPO (NZ)

*Quediocafus taieriensis* (Broun 1894): 424 - SPO (NZ)

STAPHYLININA

*Hadropinus* Sharp 1889: 115

*Hadropinus fossor* Sharp 1889: 116 - NPO, East Sea (JP: HK; RU: Sakhalin)

*Hadrotes* Mäklin 1852: 313

*Hadrotes crassus* (Mannerheim 1846): 509 - NPO (CA: BC; US: AK, CA, OR, WA; MX: BN)

*Hadrotes wakefieldi* Cameron 1945b: 786 - SPO (NZ)

*Liusus* Sharp 1889: 116

*Liusus hilleri* (Weise 1877): 93 - NPO, East Sea (CN: Manchuria; JP: HN; KR; RU: Sakhalin)

*Liusus humeralis* (Matsumura 1911): 113 - NPO, East Sea (CN; JP; KR; RU: Sakhalin)

*Thinopinus* LeConte 1852: 215

*Thinopinus pictus* LeConte 1852: 216 - NPO (CA: BC; US: AK, CA, OR, WA; MX: BN)

## Habits, habitats, and classificatory notes

### MICROSILPHINAE

*Microsilpha* includes four species although others are recognized but are undescribed. Three are South American and not coastal, but the one New Zealand species, *Microsilpha litorea* Broun, is known only from seashores (Klimaszewski and Watt 1997). Only this genus is included within the subfamily.

### OMALIINAE

Worldwide, there are about 117 genera of Omaliinae placed in seven tribes (Thayer 2005). Only the genera *Crymus*, *Omaliomimus*, *Macralymma*, and *Giulianium* seem entirely restricted to seashores. Some species within the genera *Micralymma* and *Omalium* seem restricted to seashores. All of the coastal species thus far described occur at high latitudes and not in the tropics.

### APHAENOSTEMMINI

*Giulianium* includes three species, all of them found under debris below the high tide mark, of beaches of the North Pacific Ocean. Larvae are unknown and there is no known association with seaweed (Ahn and Ashe 1999).

### OMALIINI

*Crymus* (= *Arpediomimus*) includes two species, *Crymus antarcticus* and *Crymus kronii*, both associated with seaweed on seashores (Steel 1964). The larva of *Crymus kronii* was described by Steel (1964). Hughes et al. (2004) pointed out the existence of this species in the intertidal zone, not just on the South Island of New Zealand, but also on the Antipodes, Auckland, and Campbell islands of New Zealand, and found that it is one of the hosts of *Cucujomyces phycophilus* Weir and Rossi (Ascomycetes: Laboulbeniales).

*Macralymma* includes only one species, *Macralymma punctiventre* Cameron, and it is precinctive (“endemic”) to New Zealand. Cameron (1945c) found the specimen in the Broun collection, labeled ‘Taieri Beach.’ It is widespread on the South Island of New Zealand and also occurs on Chatham Island. It is found under rotting kelp on sandy beaches (Emberson 1998). Hughes et al. (2004) pointed out the existence of this species in the intertidal zone of the Antipodes Islands of New Zealand and found that is one of the hosts of *Cucujomyces phycophilus*.

*Micralymma marinum* adults and larvae inhabit cracks in rocks in the intertidal zone of rocky coastlines. Adults and larvae are predacious, but their prey range is uncertain (Thayer 1985), probably including Collembola. In Britain, adults overwinter and larvae develop during the summer months (Steel 1970), or adults and larvae overwinter (King et al. 1979). In the northeastern USA some of the beetles may overwinter as larvae (Thayer 1985). These apterous beetles tolerate immersion in seawater (Elliott et al. 1983). A second species, *Macralymma brevilingue* Schiødte (1845: 377, syn. *Macralymma dicksoni* Mäklin 1878: 24) has similar habits but is not entirely restricted to seashores, being also found in damp moss near coasts (Steel 1958) so we do not list it. A third species, *Macralymma laticolle*, was admitted by Motschulsky (1860), its describer, not to belong to the genus *Micralymma*, but has not yet been assigned to another genus (see also Steel 1958). A fourth species, *Macralymma caucasicum* Melichar, is not coastal (Steel 1962).

*Omaliomimus* occurs only on seashores, and some species may be abundant in rotting seaweed (Steel 1964). The larva of *Omaliomimus venator* was described by Steel (1964); Hughes et al. (2004) pointed out the existence of this species in the intertidal zone not just on the New Zealand mainland, but also on the Antipodes, Auckland, Campbell, Macquarie and Snares islands of New Zealand, and found that is one of the hosts of *Cucujomyces phycophilus* Weir and Rossi (Ascomycetes: Laboulbeniales). Emberson (1998) pointed out the existence of undescribed species of *Omaliomimus* from the Chatham Islands of New Zealand.

*Omalium* species occupy various habitats, and only some occur on seashores associated with drifted seaweed. Seashore species include the European *Omalium laeviusculum*, *Omalium riparium*, and *Omalium rugulipenne*, as well as the North American *Omalium algarum*. Backlund (1945) found *Omalium laeviusculum* exclusively in deep layers of seaweed beds, but *Omalium riparium* was also found in carrion. Mjöberg (1906) described the pupa of *Omalium riparium*, and noted that it took seven days to develop to the adult. Steel (1970) found that larvae of *Omalium laeviusculum* and *Omalium riparium* occur in the summer months in Britain, whereas Larsson and Gigha (1959) had reported larvae of the latter in the winter in Iceland. Populations of *Omalium riparium* inhabiting Mediterranean shores have smaller adults than do those from northern Europe and have been considered a distinct subspecies (*Omalium riparium impar* Mulsant & Rey). *Omalium littorale* Kraatzseems to be strictly a seashore species in northern Europe, but in southern Europe it has been reported from high altitudes far from the sea (Zanetti 1987), so is not included in the checklist.

### PSELAPHINAE

Worldwide there are 1200 genera of Pselaphinae, and all are predacious (Thayer 2005). Seashores are a very minor part of their habitat range. The genus *Physoplectus* is known only from saline coastal habitats.

### BATRISITAE BATRISINI

*Arthromelus quadratus*, *Batriscenites celer*, *Batriscenites humicola*, and *Batrisocenus foveiterminalis* adults were all found in mangrove forests in Singapore, where most of them were associated with mounds of *Thalassia* (Hydrocharitaceae) turtlegrass (Tanokuchi 1989).

### GONIACERITAE BRACHYGLUTINI

*Berlara bella* adults were collected in a mangrove forest in Singapore, but not in parts inundated by the tide (Tanokuchi 1989).

*Brachygluta* has at least 43 species, but just six of them, known from America north of Mexico, appear to be restricted to coastal habitats, all these on the Atlantic coast (Chandler 1997).

*Briaraxis depressa*, the only representative of this genus, is known only from “under rubbish or logs on the beach” in the circum-Caribbean regions (Chandler 1992, 2002).

*Mangalobythus furcifer*, *Mangalobythus acutifolius*, and *Mangalobythus murphyi* adults were all collected in mangrove forests in Singapore or Thailand, where adults were seen to be active on open ground at low tide (Tanokuchi 1989).

The genus *Nisaxis* appears to be entirely coastal. *Nisaxis maritima* is known only from coastal habitats in United States Gulf Coast States; other species have been found in coastal saline habitats and also in inland saline habitats (Chandler 1997).

*Pedisinops regulus* adults were collected in the intertidal zone and on a coral reef in Japan’s Ryukyu Islands (Sawada 1991).

The genus *Physoplectus* is entirely coastal. *Physoplectus vinsoni* is known only from coastlines in Mauritius in the Indian Ocean, whereas *Physoplectus reikoae*, *Physoplectus miyakei*, and *Physoplectus irritans* are from Pacific coastlines.

*Prosthecarthron sauteri*, originally described from Taiwan, was found to be widespread on patches of halophilous grasses close to the sea in North Korea, on patches of a reed on mud in river estuaries in the Japanese mainland, in mangrove habitats in the Ryukyu Islands, and under stones on the muddy ground of a mangrove seashore in Vietnam (Nomura et al. 2006).

### ALEOCHARINAE

Worldwide, over 1,151 genera of this subfamily have been described, but its true diversity is without doubt much greater (Thayer 2005). It now contains some 12,851 species, but Hammond (1975) postulated that it might contain as many as 100,000 species. The genera are currently distributed among 51 tribes whose relationships require much study. It contains many specialist seashore inhabitants, not only at the level of genus, but even (as currently defined) at the level of tribe. Nowhere else among the Staphylinidae have entire tribes specialized to inhabit coastal habitats.

Prepupae of Aleocharinae spin a silken cocoon in which they pupate; some earlier authors incorrectly supposed the dorsal abdominal gland to be the source of the silk, although that gland produces defensive chemicals; the cocoon is not a special adaptation to immersion in water (Frank and Thomas 1984a).

### ALEOCHARINI

*Aleochara*, the type genus of this tribe, appears to be the only one with coastal representatives, and thus far 16 are known. Its larvae develop as ectoparasitoids of cyclorrhaphous dipteran pupae within the dipteran puparium. On sea beaches, such dipteran puparia are typically found in piles of decaying seaweed, but also in carrion. All eight members of the subgenus *Emplenota* are found associated with such materials on sea beaches in Europe, north Africa, Korea, Japan, and both coasts of North America (Klimaszewski 1984). Additionally, *Aleochara (Coprochara) sulcicollis* is also found in such habitats on the Pacific coast of North America and Chile in the south Pacific; *Aleochara (Coprochara) squalithorax* is found on the Pacific shores of Japan and Korea; and *Aleochara (Coprochara) salsipotens* is found on the African shores of the Indian Ocean and the African shores of the south Atlantic. Other members of the subgenus *Coprochara* do not, or seldom, occupy beaches. Two species of the subgenus *Polystomota* (*Aleochara punctatella* and *Aleochara grisea*) occupy European shores of the North Atlantic. *Aleochara punctatella* has long been confused with *Aleochara grisea*, so old records of *Aleochara grisea*, doubtless including some in the checklist, are in doubt and need re-evaluation. Three members of the subgenus *Triochara* occupy Pacific shores of eastern Asia, no others are known, and the habitat of all three appears to be seaweed. Parasitoidism by *Aleochara (Emplenota) obscurella* (as *Aleochara algarum*) of a dipterous puparium [*Orygma luctuosum* Meigen (Diptera: Coelopidae)] was reported by Scott (1916), and then in greater detail by Scott (1920) who noted that hosts were *Coelopa pilipes* Haliday and *Coelopa frigida* F. [as *Fucomyia gravis* Haliday (Diptera: Coelopidae)]. Lesne and Mercier (1922) were able to rear a 2nd instar of *Aleochara obscurella* (as *Aleochara algarum*) from *Coelopa* puparia and described and illustrated it. Paulian (1938b) reared *Aleochara obscurella* (as *Aleochara algarum*) from ‘*Fucella fucorum* Haliday’, which we suspect referred to *Fucellia fucorum* Fallén (Diptera: Anthomyiidae), and added further descriptions and sketches of parts of the larvae; Paulian (1941) also described the larvae without specifying the host. Cals (1964) encountered *Aleochara obscurella* (as *Aleochara algarum*) as a parasitoid of *Coelopa frigida* and illustrated the pharate adult within its host puparium. Because parasitoidism is the only known way of life among larvae of at least 20 species of *Aleochara*, it is thought that all species have this habit (Peschke and Fuldner 1977; Klimaszewski 1984). Adult *Aleochara* are predacious.

### ATHETINI

*Acticola falkandica* is the sole representative of this genus. The type specimen was collected in December 1914 in seaweed at Port Stanley, Falkland Islands (Cameron 1944b).

*Adota* has three Nearctic representatives (*Acticola colpophila*, *Acticola gnypetoides*, and *Acticola maritima*) reported from seaweed stranded on Pacific shores, and three Palearctic species (*Acticola madida*, *Acticola magnipennis*, and *Acticola ushio*) from eastern Asia (Gusarov 2003b). In the British Isles, *Acticola maritima* was reported by Easton (1971, under the synonym of *Atheta immigrans*) as an adventive species. Thus all six species are known only as seashore inhabitants.

*Atheta*,at the time of the Coleopterorum Catalogus (Scheerpeltz 1934), was a generic name applied to many confused and disparate groups. Over the subsequent years monophyletic groups have been split off from it as distinct genera, but the task is not yet complete. The following six seashore species are still assigned to it, within several subgenera. *Atheta novaescotiae*,not assigned to a subgenus, is a salt-tolerant, coastal, beach-drift species known from Atlantic Canada (Klimaszewski et al. 2006). *Atheta (Actophylla) varendorffiana* is known from the North Sea coast of Germany. *Atheta (Badura) ririkoae* dwells on the coasts of Korea and Honshu, Japan, as does *Atheta (Badura) tokiokai* (but this latter also on the coasts of Kyushu). *Atheta (Datomicra) acadiensis*, from Canadian Maritime Provinces, is typically found in dry beach-drift material at the top of the littoral zone, the material consisting of dead *Ascophyllum nodosum* (L.) and *Fucus vesiculosus* L. (Klimaszewski and Majka 2007). Finally, *Atheta (Sipalatheta) algarum* is found in seaweed on the coast of Hong Kong in the South China Sea (Pace 1999b).

*Brundinia* was initially described as *Homalota meridionalis* with “variety” *marina* by Mulsant and Rey (1853). The “variety” was later recognized as a valid species, and the generic name *Brundinia* was introduced later. This, or these, species (now *Brundinia meridionalis* and *Brundinia marina*) were initially reported from plant debris in a salt marsh at Hyères, on the Mediterranean coast of France. It or they (no others have been recognized) were later reported from similar habitats on the coasts of the North Atlantic, Irish, North, and Baltic seas. On the German shores of the Wadden Sea (separated by barrier islands from the North Sea), *Brundinia marina* is a common species in the lower salt marsh, and it shows time-varying abundance within the elevational gradient (Irmler and Heller 2002).

*Halobrecta* has about seven described species which in the past have been much confused by entomologists leading to much synonymy. The species occurring on Mediterranean shores need review (Gusarov 2004). For this reason, *Halobrecta halensis* Mulsant and Rey is here listed as a distinct species. The specimens of *Halobrecta flavipes* reported by Pace (2000b) from Chaiten, Chile belong to *Halobrecta algophila* (Gusarov 2004). The specimens of *Halobrecta flavipes* reported from Inaccessible Island in the South Atlantic Ocean by Klimaszewski et al. (2002) belong to *Halobrecta algophila* (Gusarov 2004). Adults of *Exatheta cingulata* and *Exatheta consors* were described by Cameron (1920) and stated to have been collected in fungi, but Sawada (1985) transferred *Exatheta cingulata* to *Halobrecta*, and Sawada (1987) synonymized *Exatheta consors*. Because fungi are rarely found on seashores, and because all other species of *Halobrecta* occur on seashores, not in fungi, Cameron’s (1920) specimens may have been mislabeled. *Halobrecta discipula* was reported as a new species in Chile by Pace (1999a) who stated that it was found “in decaying vegetables (lettuces, onions)” near Valparaíso; if those vegetables had been dumped on a sea beach (not stated) near the port city of Valparaíso, they could have been used by the beetles as surrogates for drifted seaweed; see also comments under *Myrmecopora uvida*. Illustrations of the habitus and diagnostic structures of adult *Halobrecta flavipes* together with details about its habitat in New Brunswick are provided by Klimaszewski et al. (2008).

The synonymy of *Halobrecta flavipes* in the checklist follows Pope (1977) and Lott (2008) and differs from that given by Klimaszewski et al. (2002) following Bernhauer and Scheerpeltz (1926). Bernhauer and Scheerpeltz (1926) listed ‘*Aleochara elongatula* Stephens’ (1832) as a synonym of *Halobrecta flavipes*, but Stephens (1832) referred to *Aleochara elongatula* Gravenhorst which, if Stephens was correct in his identification, refers to what is now called *Atheta (Philhygra) elongatula* (Gravenhorst) or *Philhygra elongatula*, a species which is not closely related. Furthermore, the habitat specified by Stephens (1832) for this species [“not common: found occasionally within the metropolitan district” (of London)] does not seem a likely habitat for a species of *Halobrecta*. If ‘*Aleochara elongatula* Stephens’ really is a synonym, it is the senior name and should be listed first among synonyms; however, this is likely to be a misidentification by Stephens. Also, according to Bernhauer and Scheerpeltz (1926), *Halobrecta atricilla* (Scriba, 1866): 290 *nec* Erichson is a synonym of *Halobrecta flavipes* and is so ranked in the checklist. For unexplained reasons, Bernhauer and Scheerpeltz (1926) did not accept Scriba’s (1866) redescription of *Halobrecta atricilla* (Erichson) as pertaining to that species.

*Hydrosmecta subalgarum* is the only species assigned to this genus and is reported to inhabit seashores. Four specimens, collected under seaweed on sand at Tai Long, Hong Kong, China, are the only known collection of this species (Pace 1999b).

*Iotarphia* is monotypic. Its single species, *Iotarphia australis*, is known from adult specimens collected in a “maritime habitat” from Rockdale near Sydney and from Illawarra, both in New South Wales, Australia (Cameron 1943).

*Osakatheta* is monotypic. The single species, *Osakatheta yasukoae*, is known only from adults collected under stones on tidal flats at river mouths on the eastern coast of Honshu, Japan’s largest island, where their habitat is threatened by industrial development (Maruyama et al. 2008).

*Pontomalota* contains two species, *Pontomalota opaca* and *Pontomalota terminalis*, both of them known from the Pacific coast of North America. They inhabit the mid to upper littoral zone of fine-grained sandy beaches, covered by tides only once or twice each month and containing stranded seaweed. Adults appear to be most active at night or on heavily cloudy days. They may occasionally be very abundant with hundreds of individuals per m2. Whereas *Pontomalota terminalis* is known only from California, *Pontomalota opaca* is distributed from Alaska to Baja California and shows clinal variation in color with specimens black in the north to light brown in the south. Their immature stages are unknown (Ahn and Ashe 1992). Adults spend the daylight hours beneath driftwood or piles of stranded seaweed (Kincaid 1961b).

*Psammopora* contains only one species, *Psammopora delittlei*; adults were detected on a sandy beach in Tasmania, Australia (Pace 2003).

*Psammostiba* is a genus containing five species, all from seashores in the northern Pacific, with two species in the Nearctic (*Psammostiba comparabilis* and *Psammostiba kenaii*) and three in the Palearctic (*Psammostiba hilleri*, *Psammostiba jessoensis*, and *Psammostiba kamtschatica*). Adults (the immature stages have not been reported) have been found in drifted seaweed (Gusarov 2003b).

*Tarphiota* has three species (*Tarphiota geniculata*, *Tarphiota fucicola*, and *Tarphiota densa*) confined to fine-grained sandy seashores of Pacific North America. They live in the mid- to upper- intertidal zone containing decaying seaweed and covered by only one or two high tides monthly. Larvae are unknown (Ahn 1996b, 1999).

*Thinusa* contains only two species (*Tarphiota fletcheri* and *Tarphiota maritima*) that live in the intertidal zone of sandy beaches immersed daily by tides on the Pacific coast of North America (Moore and Legner 1977; Topp and Ring 1988a; Ahn 1997b). Adults are active at night (Kincaid 1961b).

### DIGLOTTINI

Various genera have been removed to other tribes, leaving only the genera *Diglotta* and *Paradiglotta*.

*Diglotta* contains eight species, three (*Diglotta mersa*, *Diglotta sinuaticollis*, and *Diglotta littoralis*) from the North Atlantic and adjacent seas (North, Irish, and Mediterranean), one (*Diglotta brasiliensis*) from the South Atlantic, one (*Diglotta secqi*) from the Red Sea, two (*Diglotta legneri* and *Diglotta pacifica*) from the North Pacific, and one (*Diglotta maritima*) from the South Pacific. Lohse (1985), followed by Haghebaert (1991) confused the two European species of *Diglotta*; although this issue was resolved by Good (1998), distribution records of these two species are doubtless still confused. A larva, collected on the west coast of Denmark in June 1917 in association with adults of *Diglotta mersa*, was described and illustrated by Kemner (1925). Of the two European species, *Diglotta sinuaticollis* (as *Diglotta mersa*) occupies the intertidal and supralittoral zone of sandy beaches, whereas *Diglotta mersa* (as *Diglotta submarina*) occurs mostly in salt and mud-marshes (Haghebaert 1991). The type locality of *Diglotta brasiliensis* is the intertidal zone of an estuarine beach in the state of Paraná (Caron and Ribeiro-Costa 2008). The North American species occur on sandy beaches (Moore and Orth 1979a). The adults of the western Palaearctic and Brazilian species have 4 posterior tarsomeres whereas the North American and Pacific species have 5 tarsomeres (Haghebaert 1991; Caron and Ribeiro-Costa 2008; Klimaszewski et al. 2008). Illustrations of the habitus and diagnostic structures of adult *Diglotta mersa* together with details about its habitat in New Brunswick are provided by Klimaszewski et al. (2008).

*Paradiglotta*, with its single species *Paradiglotta nunni* Ashe and Ahn (2005) from New Zealand is not included in the present list because it has not been found on seashores, only inland.

### FALAGRIINI

Falagriini are a tribe of about 30 genera worldwide, only two of which have coastal representatives. The species of America north of Mexico were revised by Hoebeke (1985). A cladistic analysis of the genera of America north of Mexico by Ahn and Ashe (1995) showed monophyly of *Bryobiota* together with *Myrmecopora*, setting them apart from other genera in this tribe, which needs further study including more terrestrial falagriines.

The two species of *Bryobiota* (*Bryobiota bicolor* and *Bryobiota giulianii*) inhabit sandy beaches of the Pacific coast of North America. They live in the upper littoral zone, which is covered by tides only once or twice each month and contains buried decaying seaweed (Topp and Ring 1988a; Ahn and Ashe 1995). Larvae and diet are unreported.

*Myrmecopora* is currently divided into three subgenera (*Lamproxenusa*, *Paraxenusa*, and *Xenusa*). Fourteen species of *Myrmecopora* have been reported from seashores. Four species assigned to subgenus *Lamproxenusa* (*Myrmecopora algarum*, *Myrmecopora chinensis*, *Myrmecopora reticulata*, and *Myrmecopora rufescens*) dwell on the shores of eastern Asia in seaweed on sandy beaches (Assing 1997b). One species assigned to *Paraxenusa* (*Myrmecopora laesa*) is known from the Mediterranean Sea and the Canary Islands of the north Atlantic. Ten species assigned to *Xenusa* range from the shores of the Red, Black, and Mediterranean seas to the north Atlantic with its adjacent seas (Irish, North, and Baltic); one of these (*Myrmecopora maritima*) is known only from Madeira and the Canary Islands, and one (*Myrmecopora bernhaueri*) only from the Red Sea; additional species occupy other habitats. *Myrmecopora tenuicornis* (Küster) was treated as a synonym of *Myrmecopora laesa* (Erichson) by Fauvel (1902) and subsequent authors. Assing (1997a) expressed misgivings about this synonymy but explained that the location of the type specimen of *Myrmecopora tenuicornis* is unknown, so the synonymy cannot be resolved. *Myrmecopora (Xenusa) uvida*, a species widespread in the western Palaearctic, was newly reported as an adventive species in Chile by Pace (1999a) who stated that it was found “under decaying vegetable products (cauliflowers, cabbages, onions, etc.)” near Antofagasta; if those vegetables had been dumped on a sea beach (not stated) near the port city of Antofagasta, they could have been used by the beetles as surrogates for drifted seaweed; see also comments under *Halobrecta discipula*.

### HOMALOTINI

Most genera and species of this tribe are not seashore inhabitants. Four genera, *Heterota*, *Paractocharis*, *Pseudopasilia* and *Thinobiosus*, are exclusively seashore species. Just one species of *Linoglossa* has been reported from coastal habitats. Two species of subgenus *Halmaeusa* Kiesenwetter (1877) [= *Antarctophytosus* Enderlein (1909) = *Paraphytosus* Cameron (1917e) = *Austromalota* Brèthes (1925)] of the genus *Leptusa* Kraatz (1856), namely *Leptusa atriceps* (C.O. Waterhouse 1875: 54 and 1879: 230) from South Georgia and Kerguelen Island, and *Leptusa darwinii* (F.H. Waterhouse 1879: 531) from the Falkland Islands and South Georgia (= *rufomixtus* Brèthes 1925: 171) are omitted from the checklist because they are not strictly seashore species (Steel 1964).

*Cameronium* has two species in the Indian Ocean (*Cameronium flavipenne* and *Cameronium gomyi*), one in the Red Sea (*Cameronium obockianus*), one in North Africa (*Cameronium liebmanni* Scheerpeltz 1957a) and, remarkably, one in the Gulf of California (*Cameronium sonorensis*). If the last is correctly assigned to genus, the dispersal of its ancestors to that locale would be surprising and not easily explicable; consequently, its placement should be reinvestigated. *Cameronium liebmanni* is known only from an inland freshwater lake in Algeria so we do not include it in the present compilation. In Somalia, *Cameronium flavipenne* adults were trapped more abundantly in the wet season than in the dry season (Chelazzi et al. 1983).

*Heterota* is represented by ten species distributed from the East Sea (*Heterota sunjaei*), South China Sea (*Heterota arenaria*), and Bali Sea (*Heterota rougemonti*) through the Mascarene Islands of the Indian Ocean (*Heterota gomyi*, *Heterota obscura*, and *Heterota vinsoni*), the Red Sea (*Heterota brevicollis* and *Heterota pictipennis*), to the Mediterranean (*Heterota pamphylica* and *Heterota plumbea*). The distribution of the last of these extends from the Mediterranean to the west coasts of Europe and the Canary Islands, and more recently was discovered in southern and northwestern Florida (USA), Jamaica, and the Caribbean coast of Mexico (Frank and Thomas 1984b). There is no apparent reason to believe that movement of *Heterota plumbea* from Europe to the Canary Islands and then to the Caribbean was assisted by humans. Small, salt-tolerant, winged insects are among the most likely candidates to disperse westward naturally with assistance of trade winds at tropical and subtropical latitudes. An annotated catalog of the species is provided by Park et al. (2008). On a sandy beach in Somalia, *Heterota pictipennis* adults were trapped more abundantly in the wet season than in the dry season (Chelazzi et al. 1983).

*Linoglossa murphyi* adults were collected in a mangrove forest in Singapore (Sawada 1991), but there is no evidence that other species of the genus are halobionts.

*Paractocharis* is represented by three species. The first described was *Paractocharis fucicola*, from sandy beaches under seaweed at Changi, Singapore, in the South China Sea (Cameron 1917c). The others, *Paractocharis deharvengi* and *Paractocharis orousseti*, were both found at Puerto Galera on Mindoro, Philippines, in the Luzon Sea, in “lavages” (washings) on the beach (Pace 1990).

*Pseudopasilia* is represented only by *Pseudopasilia testacea*, found on the coasts of the North Atlantic (England and France), North Sea (Belgium and southeastern England), and western Mediterranean (southern France, Italy including Sardinia, Tunisia, and probably Croatia). For many years entomologists confused *Arena tabida* with *Pseudopasilia testacea* (Tronquet 2003). Tronquet (2003) also noted adults found under small stones in the littoral zone.

*Thinobiosus salinus* is known only from the shores of Sonora in the Gulf of Mexico, where specimens were found in seaweed on the edge of a tidal pool (Moore and Legner 1977). No other species have been assigned to this genus.

### LIPAROCEPHALINI

All members of this tribe appear restricted to coastal habitats. It appears to be monophyletic, with relationships of its seven genera suggested as ((*Baeostethus*, *Ianmoorea*) (*Paramblopusa* ((*Amblopusa*, *Halorhadinus*) (*Liparocephalus*, *Diaulota*)))) by Ahn et al. (2010). Five of its genera appear restricted to the north Pacific Ocean (with adjoining Gulf of California and East Sea), and two to the south Pacific.

*Amblopusa* contains five species, all in the North Pacific, two of them (*Amblopusa alaskana* and *Amblopusa brevipes*) in North America, two (*Amblopusa pacifica* and *Amblopusa hokkaidona*) on Hokkaido in northern Japan, and one (*Amblopusa magna*) in the Far East of Russia. All of them inhabit the mid-littoral zone. A late instar of *Amblopusa alaskana* was described in detail by Ahn and Ashe (1996a). *Amblopusa pacifica* adults were collected in wrack at Akeshi, eastern Hokkaido, in August 1990 (Sawada 1991). *Amblopusa magna* adults were collected in piles of drifted seaweed on fine sand beaches at Ryazonovka near Slavyanka, Russia in June 1993.

*Baeostethus* has one species (*Baeostethus chiltoni*) that occupies rocky intertidal areas in the Antarctic islands of New Zealand. Adults, as is typical of Liparocephalini, are flightless; a late instar was described by Steel (1964) and by Leschen et al. (2002).

*Diaulota densissima* adults were found to be plentiful along the rocky alga-covered shores of Departure Bay (British Columbia, Canada), and larvae were found and illustrated; adults are flightless (Saunders 1928). Adults and larvae of *Diaulota vandykei*, and adults and a few suspected larvae of *Diaulota densissima* were obtained from cracks of rocks and under algae at Moss Beach (San Mateo County, California, USA) in January; brief descriptions and an illustration of the apex of the larval abdomen of both was provided by Chamberlin and Ferris (1929). Moore (1956b) added some diagnostic notes on the structures of larvae of *Diaulota densissima*, *Diaulota fulviventris*, *Diaulota vandykei*, and *Diaulota harteri* collected from beaches in California and Baja California (Mexico) and provided a key to identify them. Meyerdirk (1969) studied a population of *Diaulota fulviventris* at La Jolla (California, USA) and found adults and larvae in June, August, September, and December 1968 with the number of beetles averaging 0.70 per cm2, increasing to 1.1 per cm2 in March 1969. The range of the population extended from 0.76 to 1.83 m above mean low tide, exclusively in association with the acorn barnacle *Chthamalus fissus* Darwin (Thoracica: Chthamalidae). The barnacle provided refuge from wave force and currents. Beetle activity was highest at low tide, and ceased when the substrate became saturated with sea water. Laboratory observations showed no evidence of a circadian or tidal rhythm. Pupae were formed in silk-like cocoons within empty barnacle tests and cracks in rocks. Adults respired under water by means of cutaneous respiration, expanding their abdomen to expose more membranous area (between sclerotized plates) when they were submerged.

*Halorhadinus* is represented by three species inhabiting the littoral zone of boulder shores and sandy beaches where seaweed is stranded in Korea and Japan. Maruyama and Hayashi (2009) discussed the habitat of *Halorhadinus* in detail. Their immature stages are unknown (Ahn 2001).

*Ianmoorea* is represented by a single species, *Ianmoorea zealandica*,detected under fine gravels in the intertidal region of Breaker Bay, Wellington, New Zealand (Ahn 2004, 2006).

*Liparocephalus* is represented by four species. *Liparocephalus cordicollis* adults (misidentified as those of *Liparocephalus brevipennis*) were found to be plentiful along the rocky alga-covered shores of Departure Bay (British Columbia, Canada), and larvae were found and illustrated. Adults are flightless. Placed on the surface of a dish containing water and algae, adults descended and moved among the plants under water. They were thought to carry a film of air trapped “in the hairs of the body.” Oviposition and pupation were not obtained in the laboratory. In the laboratory, larvae ate pupae of the chironomid *Telmatogeton* Schiner, and some ate dead larvae of that genus and of *Camptocladius* Wulp. Adults were not observed to feed. The disappearance of algae from the rocks during the summer may account for disappearance of the beetles and their subsequent reappearance during the winter. The foregoing account by Saunders (1928) is confusing in that it is drawn from observations on both *Liparocephalus cordicollis* and *Diaulota densissima* and sometimes makes no distinction between the two. Topp and Ring (1988b) reinvestigated, and found *Liparocephalus cordicollis* between 0.2 and 2.0 m above lowest spring tide on rocky shores of Vancouver Island (British Columbia, Canada). Adults and larvae can withstand submergence in seawater for > 2 weeks at 10oC, and thus can withstand continuous inundation from one spring tide to the next, although submerged larvae cease feeding and growing and do not pupate. Eggs were deposited in rock crevices or between the thalli of algae. Prepupae of *Liparocephalus cordicollis* spin silken cocoons incorporating plant fragments in which to pupate. *Liparocephalus cordicollis* adults, and a few larvae and pupae, were found at Moss Beach (San Mateo County, California, USA) in November and December (Chamberlin and Ferris 1929). The final instar was described and illustrated, and the pupa was stated to be formed within a cocoon. A brief redescription by Moore (1956b) of the larva failed to clarify which of the species (*Liparocephalus brevipennis* or *Liparocephalus cordicollis*) he was describing. *Liparocephalus tokunagai* adults and larvae were found abundantly on rocky shores between high and low tidemarks in the spring of the year. The adults were seen to devour amphipod crustaceans of the genus *Gammarus* Fabricius (Sakaguti 1944). The final instar of *Liparocephalus cordicollis* was described and illustrated by Ahn (1997a) with diagnostic characters for larvae of *Liparocephalus brevipennis* and *Liparocephalus tokunagai* together with a key for identification of larvae.

*Paramblopusa* was named by Ahn and Ashe (1996a) as a new genus to contain *Paramblopusa borealis*, transferred from *Amblopusa* and known from the Pacific coast of North America where specimens have been collected under rocks on tidal flats. Later, *Paramblopusa eoa* was described from the Kuril Islands of Russia, on rocks below a cliff at Negodnaya Bay (Ahn and Maruyama 2000).

### MYLLAENINI

Concepts of the tribe Myllaenini have changed radically in the past few years, and genera such as *Bryothinusa* and *Rothium* have been transferred to it from other tribes.

*Brachypronomaea esakii* adults were found on a coral reef five km off the coast of Ishigaki Island, one of the Ryukyu Islands of southern Japan. This reef is submerged for all but about two hours daily. Adults are apterous, and the food and immature stages are unknown (Esaki 1956). Later collections from Okinawa in March–May 2002 showed that the beetles occupied coral reefs having large populations of Collembola suggesting that the beetles are predatory on the springtails (Ahn et al. 2003). The second species assigned to this genus is *Brachypronomaea sawadai*, described from the Bay of St. Vincent in New Caledonia by Jarrige (1964) without further information on its habitat. The nomenclature is not totally straightforward because Jarrige (1964) equated *Thalassopora* [a genus he described (Jarrige 1959) to include *Thalassopora nosybiana* from Madagascar, and *Thalassopora marchemarchadi* (the type species) from what is now Vietnam] with *Brachypronomaea* in a heading “*Brachypronomaea* Sawada, 1956 (*Thalassopora* Jarr., 1959)”. Nowhere in the following text did he mention this was a newly proposed synonymy. However, the text included description of a new species, *Brachypronomaea sawadai*, together with mention of characters of “*B. Marche-Marchadi*” and “*B. Esakii*”. Jarrige’s (1964) intent was clearly to synonymize *Thalassopora* with *Brachypronomaea*. We believe that Jarrige (1964), despite lack of discussion, made a formal synonymy of *Thalassopora* with *Brachypronomaea* thereby transferring both the species he had originally placed in *Thalassopora*. A revision of the four species (*Brachypronomaea esakii*, *Brachypronomaea marchemarchadi*, *Brachypronomaea nosybiana*, and *Brachypronomaea sawadai*) is necessary. The habitat of *Thalassopora* (now *Brachypronomaea*) *nosybiana* was described as in sand and rocks near the oceanographical station of Nosy Bé, Madagascar (Paulian 1959). Its final instar (recognized because it was with adults, not reared) was described and illustrated, its digestive tube being packed with unicellular algae (Paulian 1959).

*Bryothinusa*, with 30 described species, restricted to seashores and with no species from other habitats, has the greatest diversity of all genera of the coastal Aleocharinae. Twenty-four live on shores of the Pacific Ocean and its surrounding seas, two (*Brachypronomaea madecassa* and *Brachypronomaea perexilis*) on the shores of the Indian Ocean, and four (*Brachypronomaea cameroni*, *Brachypronomaea peyerimhoffi*, *Brachypronomaea subtilissima*, and *Brachypronomaea testacea*) on the shores of the Red Sea (one of these also found in the Mediterranean). None has been reported from the Atlantic Ocean. Revisions have been published by Pace (1986), Haghebaert (1995), and Ashe (2005). Adults of *Brachypronomaea sakishimana* were collected in a mangrove forest (Sawada 1991), and those of *Brachypronomaea chengae* at light (Ahn 1998). The larva of the Californian *Brachypronomaea catalinae* was described by Moore and Orth (1979b) who described its habitat as “a shallow reef which is exposed at low-water and under water at high tide ... largely a field of boulders two or three feet across with smaller stones and gravel in sand ... adults and larvae were found beneath and on the stones in an association with dense worm tubes, chitons, limpets, small abalones, flatworms, small crabs and brittle stars.” Four species (*Brachypronomaea gangjinensis*, *Brachypronomaea koreana*, *Brachypronomaea minuta*, and *Brachypronomaea nakanei*) have been found on Korean shores (Ahn and Jeon 2004).

Of the *Bryothinusa* species found in Hong Kong, the habitats were distinguished as follows by Moore and Legner (1971) and Moore et al. (1973): (*Brachypronomaea chani*): “At low water they wander on the surface of the mud flat and at high tide they are under seawater by holding tightly to rocks and dead shells”; (*Brachypronomaea sawadai*): “Found among rock crevices and shells of barnacles and oysters. When burrowing in the sand never more than 1 cm deep. Can be found throughout the year.”; (*Brachypronomaea sinensis*): “Found among rocks, wandering on sand or burrowing in the sand as deep as 36 cm. Seems to prefer a sandy beach. Can be found throughout the year.”; (*Brachypronomaea hongkongensis*): “Found wandering on sand or burrowing in sand as deep as 36 cm from mid-tidal zone to low tidal zone. Specimens were found from February to April” (Moore and Legner 1971; Moore et al. 1973). Descriptions do not suggest that members of the genus are alike in sharing one kind of habitat. The habitat of *Brachypronomaea fluenta* was even more unusual: it was “beneath the surface of a fresh water stream a short distance from the seashore...[it] seems to have invaded a fresh water habitat directly from salt water” (Moore and Legner 1975). Adults of four of the Hong Kong *Bryothinusa* species (*Brachypronomaea chani*, *Brachypronomaea hongkongensis*, *Brachypronomaea sawadai*, and *Brachypronomaea sinensis*) were apparently able to breathe by plastron respiration, to feed on decaying microcrustacea, to occupy the mid-intertidal zone (0.6–1.5 m above mean tide level), to spend their time mainly at 0–15 cm below the sand surface, to mate on the sand surface, to occupy sand of low organic content, and to be macropterous except for *Brachypronomaea chani* (Wong and Chan 1977). Larvae of these four remained below the sand surface. The larva of *Brachypronomaea koreana* was described by Jeon and Ahn (2009), making it only the second species of the genus so treated (with the larva identified by mitochondrial DNA).

*Corallis polyporum* adults were stated by Fauvel (1878a) to be “sous-marines” (submarine or aquatic in the sea) under coral polyps in March. They occur together with those of *Polypea*, both genera being monotypic (Fauvel 1878a).

*Lautaea murphyi* adults were collected under mangroves in intertidal mud flats. Their digestive tracts were filled with fragments of harpacticoid copepods (Sawada 1989a). The genus is monotypic.

*Myllaena* is a genus distributed worldwide whose members are typically found in freshwater habitats, such as on the banks of lakes and streams, and in swamps. However, the North American *Myllaena insipiens* Casey seems to have been collected only at or very close to coasts of the Atlantic and Gulf of Mexico of the USA (Klimaszewski 1982a), and is provisionally included in this list. *Myllaena leleupi* Pace (1985) is excluded from the checklist because its type locality in the Galapagos Islands is 4 km from the coast, despite a later record in a coastal habitat (Klimaszewski and Peck 1998).

*Polypea corallis* adults were said to have been found in the ocean under coral polyps, in March, to occur with those of *Corallis*, and to be submarine (Fauvel 1878a). Klimaszewski (1982b) argued that *Polypea* seems to lack any structural modification for a submarine existence.

*Rothium* was first included in Myllaenini by Ahn and Ashe (1996c). Its six described species inhabit the Pacific (including Gulf of California) coasts of North and South America. The first to be described, *Rothium sonorensis*, was collected in the intertidal zone “from algae covered pitted ryolite” and from “a tide pool” (Moore and Legner 1977). Discovery of two further Mexican species, and three from parts of Ecuador (including the Galapagos Islands) and Peru yielded little more information about their habitats. The larva of *Rothium sonorensis* was described by Moore (1977) and subsequently redescribed in detail by Ahn and Ashe (1996c).

### OXYPODINI

Seashore representatives of this large tribe belong to seem to be only four genera, all monotypic: *Chilodera*, *Dasydera*, *Gyronotus*, and *Oreuryalea* (Assing and Maruyama 2002).

*Chilodera falklandica* is known only from seaweed at Port Stanley in the Falkland Islands in the South Atlantic, where specimens were collected in December 1914 (Cameron 1944b).

*Dasydera algophila* was found among seaweeds on Mokohinau Island, New Zealand (Broun 1886).

*Gyronotus rufipennis* is known only from North Island, New Zealand (Broun 1880).

*Oreuryalea watanabei* is known from the Russian Far East and northern Japan, where it was detected among drifted seaweed and other debris on beaches. Four female specimens collected in July and September each had a single mature egg in its ovaries (Assing and Maruyama 2002).

### PHYTOSINI

The relationships of the tribe are unclear, and it is not yet certain that the tribe is monophyletic. The four genera remaining in this tribe, after removal of others to Myllaenini etc., are *Actocharis*, *Arena*, *Euphytosus*, and *Phytosus*.

*Actocharis* contains only two European species, *Actocharis readingii* and *Actocharis cassandrensis*. Both occur on Mediterranean coasts, and the first also occurs on North Atlantic coasts of England and France.

*Arena* includes one European species (*Arena tabida*) and another (*Arena fultoni*) from New Zealand, a remarkable distribution if these placements are actually correct (*Arena fultoni* needs re-evaluation). *Arena tabida* was for years confused by entomologists with *Pseudopasilia testacea* (Homalotini) but it has a narrower distribution on the Atlantic coast of northwestern France, with the coasts of England and Wales, Irish Sea coasts of England and Wales, and North Sea coasts of England, Scotland, Netherlands, Germany, and Denmark, and is associated with drifted *Fucus* (Tronquet 2003).

Bernhauer (1922a) described specimens collected by Hans Sauter at Alikang in Taiwan under the name *Phytosus (Paraphytosus) schenklingi*. No information about its habitat was provided. However, the name *Paraphytosus* Bernhauer was preoccupied (by *Paraphytosus* Cameron 1917e). In 1926, the Coleopterorum Catalogus designated *Euphytosus* Bernhauer and Scheerpeltzas a replacement name (through objective synonymy) for *Paraphytosus* Bernhauer, with *Paraphytosus (Euphytosus) schenklingi* as its type and only species.

Haghebaert (1993) excluded *Euphytosus* from the genus *Phytosus* with the consequence that it was automatically raised to generic rank; in that same work, Haghebaert also suggested (he wrote that he planned a later publication on the subject) an unnecessary new name (*Pseudophytosus*) for *Euphytosus*, so *Pseudophytosus* is not a valid name.

*Phytosus* is currently divided into two subgenera. The typical subgenus has three species; *Phytosus spinifer* in the North Atlantic and Baltic, Black, North, and Mediterranean seas; *Phytosus fenyesi* on the Atlantic coast of Senegal; and *Phytosus caribeanus* on the shores of Guadeloupe in the West Indies. All five members of subgenus *Actosus* dwell on coasts of the North Atlantic and/or adjacent seas (Mediterranean, Baltic, or North). The present checklist does not mention *Phytosus atriceps* C.O. Waterhouse (1875), from Kerguelen Island, redescribed and illustrated by him (1879). The species was made the type of a new genus, *Antarctophytosus* by Enderlein (1909). Unaware of that assignment, Cameron (1917b) reported its finding in seaweed on a sandy seashore in the Falkland Islands and made it the type of a new genus, *Paraphytosus*. Cameron (1917e) admitted that he had misidentified the specimens, and also that the generic name *Antarctophytosus* Enderlein had precedence over *Paraphytosus*. Steel (1964) considered the genera *Antarctophytosus* Enderlein (1909), *Paraphytosus* Cameron (1917b), and *Austromalota* Brèthes (1925) all to be synonyms of *Halmaeusa* Kiesenwetter (1877). Steel (1964) also found that none of the species of *Halmaeusa* is restricted to seashores.

### Incertae sedis

*Salinamexus* has two species (*Salinamexus browni* and *Salinamexus reticulatus*) that occur on the shores of the Gulf of California and one (*Salinamexus koreanus*) in Korea. Adults are able to fly. All three appear to occur exclusively under boulders or seaweed on seashores; their immature stages are unknown (Jeon and Ahn 2008). Ahn et al. (2010) showed that the genus was not a member of the Liparocephalini. Its phylogenetic position is uncertain.

### OXYTELINAE

Worldwide, there are 47 known genera in Oxytelinae (Thayer 2005).

### OXYTELINI

This tribe has about 14 genera worldwide (Newton et al. 2001), including *Anotylus*, *Blediotrogus*, *Pareiobledius*,and *Sartallus*.

*Anotylus* has about 350 species worldwide, most associated with forest leaf litter, decomposing organic matter such as dung and carrion, and in mammal or ant nests (Newton et al. 2001). However, the European *Anotylus maritimus* appears to occupy only seashore habitats, where it lives among seaweed and other drifted debris. *Anotylus speculifrons* (Kraatz 1857: 862) is often found in coastal habitats in western Europe, but occurs also in eastern Europe far from seashores; consequently we do not include it.

*Blediotrogus* has about five known species in Australia, New Zealand, and the Chatham Islands, and they are frequently found on seashores, under moist high-tide beach wrack (Makranczy 2006).

*Pareiobledius* has three known species, from the Afrotropical region, whose characteristic habitat is on seashores, under kelp (Makranczy 2006).

*Sartallus* has only one species, the Australian *Sartallus signatus*, with winged adults. That species “is common on our [?South Australian] sandy beaches, where it [the adult?] hides under the seaweed and rubbish and feeds chiefly upon dead barnacles” (Froggatt 1907).

### THINOBIINI

This is the largest oxyteline tribe with about 20 genera worldwide (Newton et al. 2001). *Carpelimus*, with several hundred species, worldwide, is one of the two largest genera. Most *Carpelimus* species occupy freshwater habitats such as the shores of rivers and lakes, and in swamps. Moore and Legner (1974b) described the larva and pupa of *Carpelimus debilis* (Casey 1889a: 374) and noted that it occurs often in stranded seaweed on the Pacific shores of North America, but occupies other habitats as well (so we do not list it). On the shores of Zanzibar, *Carpelimus lucidus* was found in seaweed and there seems to be no other habitat information, so we list it (Cameron 1944a).

*Bledius*, with over 450 species worldwide, is the other large genus within the Thinobiini. Herman (1986) classifies it according to species groups (an ordering we follow in our checklist) rather than formal subgenera. Only some *Bledius* species dwell on seashores, and some of these also occur inland in saline habitats. We attempt to list only those species that do not occur also in inland saline habitats, resulting in the inclusion of 57 species names. We refer the reader to Herman (1986: 11–72) for an excellent overview of the natural history of the genus [including its predators and parasites (partially reviewed in Frank 1982, 1985b)]. Consequently herein we mention only a few other studies.

Several *Bledius* species found on coasts have been studied in some detail and have been noted as coastal species. However, evidence suggests that these are not strictly coastal, so they are not included in the present checklist. We mention them in part lest the reader should think we overlooked them and in part because the references cited provide larval and/or behavioral descriptions that may prove useful in comparison with future larval descriptions of coastal species. Four *Bledius* species [*Bledius tricornis* (Herbst), *Bledius hinnulus* Erichson), *Bledius gallicus* Gravenhorst) as *Bledius fracticornis* (Paykull), and *Bledius pallipes* (Gravenhorst)] from Denmark have fine larval illustrations by Schiødte (1864). Five *Bledius* species [*Bledius arenarius* (Paykull), *Bledius talpa* (Gyllenhal), *Bledius subterraneus* (Erichson), *Bledius fuscipes* (Rey), and *Bledius opacus* (Block)] from Finland have fine larval descriptions and illustrations by Krogerus (1925). Four *Bledius* species [*Bledius spectabilis* Kraatz, *Bledius unicornis* (Germar), *Bledius furcatus* (Olivier), and *Bledius fuscicornis* Cameron] from France have larval descriptions by Paulian (1938a, 1941). The larva of *Bledius albonotatus* Mäklin from California was described (as *Bledius ornatus* LeConte) by Moore and Legner (1974a). Notes on behavior and development of *Bledius spectabilis* were published by Paulian (1942). Detailed studies by Bro Larsen (1936, 1951, 1952) of beetles of some Danish salt marsh and dune habitats dealt with several *Bledius* species particularly *Bledius spectabilis*. More recently, the distribution, behavior, and physiology of *Bledius spectabilis* was studied in Norfolk, England (Evans et al. 1971; Wyatt 1982, 1986; Wyatt and Foster 1988). The latter show the remarkable development of subsocial behavior among *Bledius* in which adults care for their brood. They also illustrate the form of the tunnels in which the entrance is narrowed to a bottle-neck so that it is not easily flooded by the tide (and can be blocked by the adults within a few minutes) to trap oxygen inside. Included is information on oxygen consumption and the minute algae that are the food of adults and larvae.

*Bledius fenyesi* and *Bledius monstratus* dwell on the Pacific coasts of North America in decaying, sand-covered piles of seaweed (Herman 1986). Evans’s (1980) claim that adults of *Bledius monstratus* are predacious requires verification given that other members of the genus feed on algae.

*Bledius subniger* dwells on the sandy shores of Ostvoorn (Netherlands) just below the high tide mark, tunneling within the topmost 5 cm of sand, and occurring at densities up to 500 per m2. It feeds on Chlorophyta, Cyanophyta, and Chrysophyta (diatoms). It is very active at the end of June and the beginning of July, with individuals walking on the sand surface, followed by swarming for mating or dispersal (Hollander and van Etten 1974; Hollander 1983). These authors also discuss the habits of *Bledius arenarius*, which dwells in dunes above the beach and is thus excluded from our list of seashore staphylinids.

Griffiths and Griffiths (1983) found that *Bledius punctatissimus* attained densities of up to 2,260 adults per m2 on a sheltered marine beach at Pawley’s Island (South Carolina, USA). Adults and larvae below the sand surface are regularly immersed by the tide. At low tide they emerged to form galleries just below the sand surface and fed on diatoms. This activity lasted as long as 11 hours at higher shoreline elevations or 7 hours at elevations nearer to the lower limits of its distribution. At other times, adults and larger larvae singly occupied individual deeper burrows, whereas some females occupied maternal burrows together with their eggs and small larvae in side galleries; such burrows retained air during immersion by the tide. Griffiths and Griffiths’ (1983) account is accompanied by drawings of egg, larva, pupa, and adult.

*Teropalpus* contains nine species, all with seashore distribution (Newton et al. 2001). Just one (*Teropalpus lithocharinus*) is native to the northern hemisphere, the remaining eight to the southern hemisphere, but one of the latter (*Teropalpus unicolor*), native to New Zealand, Australia, and South Africa, is adventive in Britain and arrived there before 1900. Specimens are found on seashores under driftwood and algae (Makranczy 2006).

*Thinobius* includes nearly 100 species worldwide (Newton et al. 2001). Most appear to live on banks of streams and ponds and other freshwater habitats, but a few live on intertidal mudflats. Among the latter, are *Teropalpus frizzelli* on the Pacific coast of North America, *Teropalpus marinus* in Singapore, and *Teropalpus kuroshio* on Honshu, the largest island of Japan. Dense populations of *Teropalpus frizzelli* were reported from felted growth of an alga [*Lyngyba semiplana* (C. Agardh) J. Agardh (Oscillatoriaceae)] in the intertidal zone of Willapa Bay in southwestern Washington State, where the individuals retreated into burrows among the algae when the tide was high; adults are winged, but the wings are of reduced size; adults are thought to eat “detritus”; larvae received a cursory description from Kincaid (1961a). In contrast, *Teropalpus kuroshio* adults were found in decaying drifted seaweed on a pebbly beach (Sawada 1971c), and *Teropalpus marinus* adults were found at Changi, Singapore, on sandy beaches under seaweed (Cameron 1917d). The synonymy of *Yosiityphlus* with *Thinobius* was documented by Gusarov and Makranczy (2004).

### SCYDMAENINAE CEPHENNIINI

Worldwide, over 4600 species in some 82 genera of this subfamily have been recorded (O’Keefe 2005). *Cephennodes araiorum*was found under stones on a stony/ sandy beach on the Pacific coast of Honshu, central Japan. It is the only member of the genus known from seashores, the others having been found in leaf litter and rotten deciduous wood at inland sites (Jałoszyński 2003).

### PAEDERINAE

About 221 genera of Paederinae occur worldwide; the tribal classification is under revision by L.H. Herman, so subsequent classification may be changed, and the placement of coastal species in the tribe Paederini (below) is uncertain (Thayer 2005).

### PAEDERINI

About 200 genera of Paederini occur worldwide. The monotypic genus *Chetocephalus* is based on *Chetocephalus maritimus*, which was found in seaweed on Mauritius (Cameron 1944a).

*Medon* contains about 350 species worldwide, few of them occurring on seashores. Two exceptions are *Medon marinus*, which was found in seaweed on Mauritius, and *Medon pocoferus* found on the shores of the Mediterranean Sea and the North Atlantic Ocean. Two others are *Medon prolixus* and *Medon rubeculus*, both initially described from Japan, the former first reported from seaweed at Iwosima and Amakusa (Sharp 1874), but the latter was subsequently detected in Hong Kong where it appears to be restricted to the seashore (Rougemont 2001).

*Sunius* has about 125 species worldwide, but few of these are typically found on seashores. These exceptions are *Sunius ferrugineus* from the Caribbean Sea and *Sunius minutus* from Florida. Both live in drifted seaweed, and they may represent only a single species.

*Ophioomma*, a genus only recently transferred to Paederini, has only one species, *Ophioomma rufa*. Specimens were initially collected on the Gulf of Mexico coast of Florida, by sifting debris on the beach of Charlotte Harbor.

### STAPHYLININAE

This subfamily contains 320 genera worldwide (Thayer 2005). Adults and larvae are predacious.

### STAPHYLININI

This tribe contains 200 genera worldwide (Newton et al. 2001). Those with coastal species are *Thinopinus*, *Hadrotes*, *Hadropinus*, *Liusus*, *Thinocafius*, *Cafius*, *Remus*, *Phucobius*, and *Orthidus*, which do not form a monophyletic group.

**PHILONTHINA** are a subtribe with five genera (*Thinocafius*, *Cafius*, *Phucobius*, *Remus*, and *Orthidus*) that appear to be restricted to seashores. Additionally one species each of *Bisnius*, *Gabronthus* and *Philonthus* are also confined to coastal habitats. The systematic position of *Thinocafius* is unclear and requires study in relation to the phylogeny of *Cafius*.

*Bisnius* is moderately large genus with global distribution. Only one species (*Bisnius macies*) appears to typically be found in drifted seaweed. It was originally described from Japan and was recently detected in Korea (Cho 2008).

*Cafius*, in its early sense included also species now assigned to *Remus*. An attempt by Koch (1936) to divide it into subgenera was not completed for all species known at that time, and perhaps for that reason was not followed by some later authors. Later, Coiffait (1963, 1974) elevated one subgenus (*Remus*) to generic rank and proposed a new subgeneric name (*Suborthidus*) to include one species within the remaining species of *Cafius*. Coiffait’s effort to subdivide *Cafius* applied only to European, North African and Middle Eastern species, and was similarly not followed by some later authors. A global revision of the genus is necessary. In the present study 44 species are listed within *Cafius* (sensu stricto), making this the most species-rich genus of coastal staphylinids. One seemingly unusual species (*Cafius splendoris* Last 1987) is reported from Mt. Amigwiwa 1000–2300 m in Papua New Guinea and is excluded from our list. It may be misassigned to genus. Four species are attributed to *Remus* (see below). The zoogeographic distribution of species is as follows: Pacific Ocean 29, Atlantic Ocean 8, Indian Ocean 5, Pacific plus Atlantic 1, Pacific plus Indian 1.

The larva of the European *Cafius xantholoma* was described by Paulian (1941). Larvae and pupae of the Pacific species, *Cafius canescens*, *Cafius lithocharinus*, *Cafius luteipennis*, and *Cafius seminitens*, were described by James et al. (1971). Subsequently Moore (1975) described the larva of *Cafius sulcicollis*, although Orth and Moore (1980) stated that it was actually the larva of *Cafius bistriatus*. More recently, Jeon and Ahn (2002, 2007) used DNA sequence data to associate field-collected larvae with identified adults, and were able thus to identify and describe larvae of *Cafius histrio*, *Cafius mimulus*, *Cafius rufescens*, *Cafius fucicola*, *Cafius nauticus*, and *Cafius vestitus* from the Pacific. The European *Cafius xantholoma* is the best investigated species. Backlund (1945) compared the attractiveness of sterile leaves of various flowering plants versus sterile *Fucus* fronds and found that 26 adult specimens chose *Fucus* versus only four that chose other leaves. *Cafius xantholoma* is predacious as adult and larva, and is stenotopic in deep layers of seaweed beds (Backlund 1945). Observations of *Cafius xantholoma* larvae throughout the year (Backlund 1945; Egglishaw 1965) suggest that the species is multivoltine. Adults of *Cafius xantholoma* are highly resistant to wetting and may take flight directly from the water surface (Backlund 1944), and adults of *Cafius bistriatus* show the same ability to avoid wetting and drowning (Frank et al. 1986). On the Pacific coast, mass flights of beetles including *Cafius luteipennis* have been observed and generally are in a direction parallel to the shore (Leech and Moore 1971; Evans 1980). We are unaware of reports of brachyptery among *Cafius* species. The distribution of *Cafius* species along the coast of the United States from New Jersey south to Florida and the Gulf of Mexico is determined by the abundance of drifted algae (not drifted seagrasses or marshgrasses), and some algae, for example *Sargassum* *fluitans* Borgessen, appears not to provide suitable habitat (Frank et al. 1986). The wider distribution of species originally described from the West Indies is still not determined although Frank (1985a) reported *Cafius caribeanus* from the South American mainland, and *Cafius subtilis* from Florida (USA). There are also questions about the relationship between *Cafius subtilis* and *Cafius aguayoi* (described from Massachusetts, USA), and of these to the European species now known as *Remus sericeus*. A report of *Cafius xantholoma* from Chile was later shown to be based on misidentification. Most species of *Cafius* are found on the Pacific Ocean shores, and only one species (*Cafius bistriatus*) occurs both there and on the Atlantic. It is believed to have colonized Atlantic coasts from the Pacific, and the two populations have been distinguished morphologically at the subspecific level (Frank et al. 1986). On a sandy beach in Somalia, adults of three *Cafius* species (*Cafius fonticola*, *Cafius nauticus*, and *Cafius raggazzii*), were trapped more abundantly in the dry season than in the wet, and were more active at night in the dry season than in the wet (Chelazzi et al. 1983).

*Gabronthus* was recognized as a genus distinct from *Philonthus* in 1955. It contains over 30 species; only one of these, *Gabronthus maritimus*, found in the Mediterranean, the Red Sea, the Indian Ocean, and the South China Sea, appears to be restricted to coastlines. A report of *Gabronthus maritimus* from Cuba is highly questionable and without voucher specimens so has not been accepted in this study.

*Orthidus* contains only one species (*Orthidus cribratus*) found on the Mediterranean coasts of southern Europe and northern Africa, and Atlantic coasts from Brittany (France) south to Morocco. It dwells under rocks and in piles of drifted algae on seashores (Coiffait 1974).

*Philonthus nudus* was for many years misattributed to *Cafius*, perhaps because of its atypical (for the genus *Philonthus*) coastal habitat. Its populations are distributed on the coasts of Korea, Japan, and the Kuril Islands of Russia, to the Pacific coasts of Canada and the United States, typically in drifted seaweed. *Philonthus* is a genus of over 1,000 species.

*Phucobius* contains eight species, of which seven are found under seaweed on beaches. The eighth (*Phucobius africanus*) was reported from a single specimen collected at 1600 m in western Usambara, Tanzania. The collection locality has been confused, or perhaps the speciesbelongs to another genus. Six species inhabit shorelines of the western Pacific (including the East Sea, South China Sea, and Java Sea, but not farther north than Japan), and one is found the shores of the Indian Ocean. Sharp (1874) recorded *Phucobius simulator* as common under seaweed at Amakusa and Iwosima, Japan.

*Remus* contains four species, all found in drifted seaweed on coastlines. One species (*Remus sericeus*) occurs on coasts of the North Atlantic, Baltic, Irish, North, Mediterranean, and Black seas and, remarkably, in Australia. Another species (*Remus filum*) is confined to the coasts of the Mediterranean and Black seas. Another species (*Remus pruinosus*) is known from coasts of the North Atlantic, North, and Mediterranean seas. Another species (*Remus corallicola*) is known from the South Pacific, South China, and Java seas, the Indian Ocean, and the Red Sea. The larva and pupa of *Remus sericeus* were described and illustrated by Paulian (1941). Bierig (1934a) did not record *Remus pruinosus* from Cuba as listed by Peck (2005). On a sandy beach in Somalia, adults of two *Remus* species (*Remus corallicola* and *Remus filum*), were trapped more abundantly in the dry season than in the wet (Chelazzi et al. 1983).

*Thinocafius* contains only one known species, *Thinocafius insularis*,which is known only from the Chatham Islands east of New Zealand.

**QUEDIINA** are a subtribe containing one genus (*Quediocafus*) that appears to be restricted to seashores. One or more members of two other genera (*Heterothops* and *Quedius*) may or may not be restricted to seashores.

*Heterothops* is a widespread genus. Only one species, theNorth American *Heterothops asperatus*, appears to be confined to Pacific seashores. The European *Heterothops binotatus* (Gravenhorst) is often but not exclusively found on seashores and so is not included.

*Quediocafus* is known only from New Zealand, and all three species appear confined to sea beaches. Marris (2000) mentioned that *Quediocafus insolitus* was found “under tussock mats and among coastal vegetation”.

*Quedius* is a large and widespread genus apparently without any coastal specialists. *Quedius simplicifrons* Fairmaire (1861), which has several synonyms and is widely distributed at and near the coasts of western Europe and adjacent islands, does not meet our definition of a strictly coastal species. *Quedius umbrinus* Erichson (1839a), which Mjöberg (1906) found to be the most abundant insect in drifted seaweed at Bohuslän in southwestern Sweden, is not included because it also occurs in inland habitats.

**STAPHYLININA** are a subtribe containing the strictly coastal genera *Thinopinus*, *Hadrotes*, *Hadropinus*, and *Liusus*, as well as genera that are not confined to seashores.

*Thinopinus pictus*, occupying sandy Pacific beaches of North America from Alaska south to Baja California, has adults that are mottled dark and pale (‘melanic’) on dark sand beaches, but pale on pale sand beaches (Malkin 1958). Malkin (1958) pointed out that adults are nocturnal, spending the day concealed, and was perplexed as to why there should be two color forms. On beaches near Goleta (California, USA), adults and larvae are present throughout the year; dissected females contained 2–3 eggs, which are laid singly beneath the sand and hatch in ~ 14 days (Craig 1970). These eggs are white and ~ 3.0 mm long and 2.2 mm wide. Larval development time was not determined, but larvae were observed at night running on the sand surface like the adults (Craig 1970). On the western shore of Vancouver Island (British Columbia, Canada), the main prey is *Orchestia californiana* (Brandt), an amphipod which spends the day in temporary burrows on the upper part of the beach and is ambushed at night by the adult beetles (Richards 1982). The activity pattern of the amphipod is adjusted to weather and tidal patterns, and on some nights it is active after midnight, whereas beetles are active soon after dark; thus, beetles often foraged when amphipods were inactive (Richards 1983). Beetles attacked 0.147 amphipods per minute and captured 9.1% of prey attacked (Richards 1984). Color photographs of a larva (15 mm long) and adults of the two color forms (16–18 mm long) were provided by Evans (1980).

*Hadrotes crassus* lives on the Pacific coasts of North America, and is found from Alaska south to Baja California. Its larva was described by Moore (1964b) and color photographs of adult (11–17 mm) and larva (14–16 mm) are provided by Evans (1980); adults and larva are nocturnal predators of crustaceans and insects. A species described from New Zealand as *Hadrotes wakefieldi* may belong to another, possibly undescribed, genus (Klimaszewski et al. 1996).

*Hadropinus fossor* is an old-world representative of the subtribe, inhabiting the shores of northern Japan and of Sakhalin Island (Russia). It makes burrows in sand under seaweed (Sharp 1889).

## Discussion

**Figure 6.**
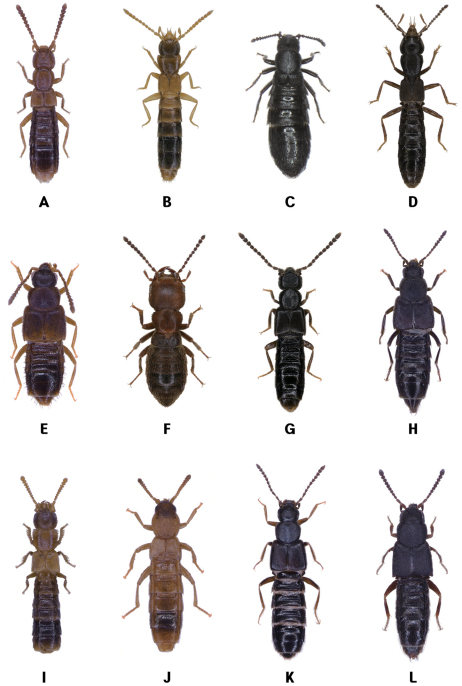
Habitus photographs. **A** *Bryobiota bicolor*, 2.8 mm **B** *Bryothinusa koreana*, 3.2 mm **C** *Diaulota aokii*, 2.6 mm **D** *Diglotta sinuaticollis*, 3.0 mm **E** *Heterota sunjaei*, 2.4 mm **F** *Liparocephalus cordicollis*, 4.3 mm **G** *Myrmecopora simillima*, 3.1 mm **H** *Oreuryalea watanabei*, 4.9 mm **I** *Phytosus balticus*, 2.7 mm **J** *Pontomalota opaca*, 3.6 mm **K** *Psammostiba hilleri*, 5.3 mm **L** *Tarphiota geniculata*, 2.3 mm.

**Figure 7.**
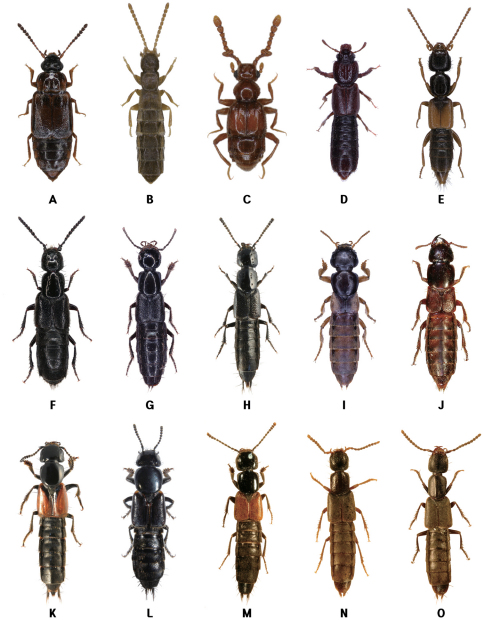
Habitus photographs. **A** *Omalium laeviusculum*, 4.3 mm **B** *Giulianium alaskanum*, 2.6 mm **C** *Prosthecarthron sauteri*, 1.9 mm **D** *Bledius fenyesi*, 4.7 mm **E** *Medon prolixus*, 4.6 mm **F** *Bisnius macies*, 8.3 mm **G** *Orthidus cribratus*, 12.2 mm **H** *Philonthus nudus*, 8.1 mm **I** *Hadropinus fossor*, 21.8 mm **J** *Hadrotes crassus*, 13.6 mm **K** *Liusus hilleri*, 15.4 mm **L** *Liusus humeralis*, 14.0 mm **M** *Phucobius simulator*, 9.8 mm **N** *Remus corallicola*, 5.5 mm **O** *Remus sericeus*, 6.8 mm.

**Figure 8.**
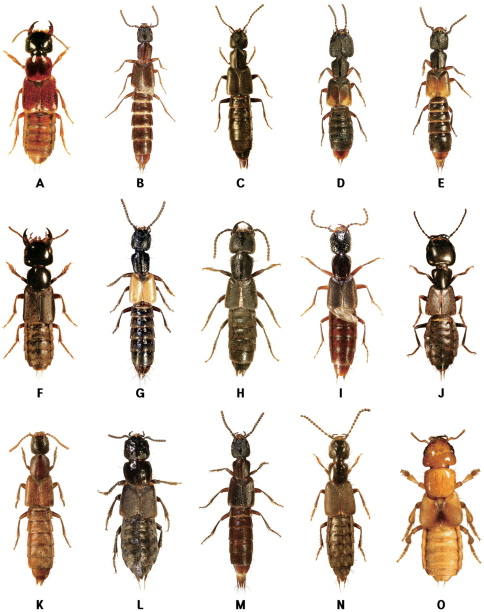
Habitus photographs. **A** *Cafius australis*, 15.4 mm **B** *C. bistriatus*, 7.7 mm **C** *C. histrio*, 8.5 mm **D** *C. lithocharinus*, male, 9.6 mm **E** *C. lithocharinus*, female, 9.3 mm **F** *C. litoreus*, 13.9 mm **G** *C. luteipennis*, 9.2 mm **H** *C. mimulus*, 8.0 mm **I** *C. pacificus*, 9.7 mm **J** *C. quadriimpressus*, 17.9 mm **K** *C. rufescens*, 6.2 mm **L** *C. seminitens*, 13.6 mm **M** *C. sulcicollis*, 7.5 mm **N** *C. xantholoma*, 7.8 mm **O** *Thinocafius insularis*, 14.3 mm.

Many kinds of insects can be found, some of them dead or dying in sea drift on shorelines. Many are winged insects that have alighted on the sea surface and have subsequently been washed up on the shore. Non-coastal species may exploit such concentrations of food. The senior author observed a pile of lawn grass clippings on a beach in Guanacaste, Costa Rica, that produced an abundance of staphylinids, none of them coastal species. Thus, would-be collectors of coastal staphylinids may be misled by the mere presence of staphylinids on seashores — a knowledge of non-coastal genera is essential. Coastal species represent only about 0.7% all Staphylinidae, although almost 400 species of them are known. Some representative species are shown in Figures 6–8.

**Figure 9.**
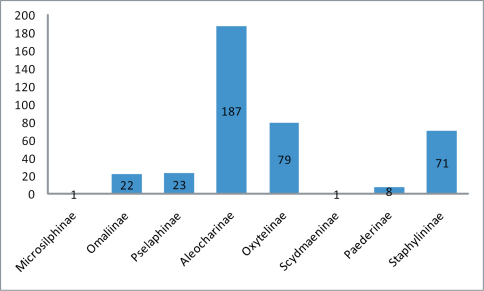
The number of coastal Staphylinidae species within each subfamily.

**Figure 10.**
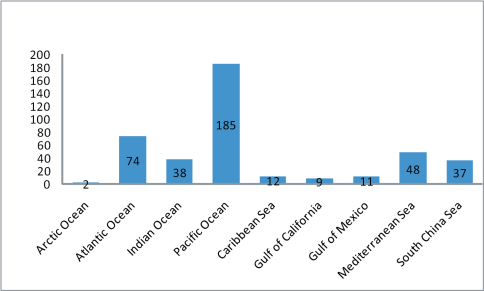
The number of coastal Staphylinidae species found in various oceans and seas.

**Figure 11.**
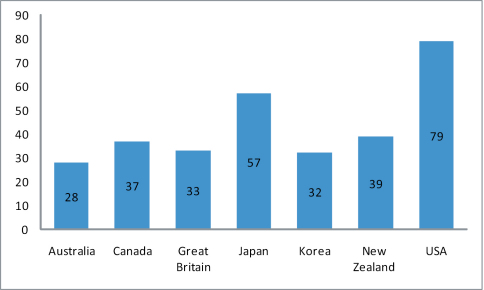
The number of coastal Staphylinidae species found in various countries.

Aleocharinae are the most species rich of the eight subfamilies with coastal representatives (Fig. 9). The Pacific Ocean has many more species of coastal staphylinids than do the other oceans (Fig. 10). The United States has more species of coastal staphylinids than do eight other countries (Fig. 11).

### Polyphyly of coastal Staphylinidae

Lawrence and Newton (2000) and Newton et al. (2001) recognized four lineages among the Staphylinidae, and included among them the Pselaphinae and Scydmaeninae (Fig. 1). All four lineages contain genera and species that are restricted to coastal habitats. Coastal genera and species are found in eight subfamilies which contain mainly non-coastal genera. No subfamily contains only coastal genera. It is not until the level of tribe that there are taxa—all of them in the Aleocharinae—that include exclusively coastal genera.

The evolution of coastal genera in the Staphylinidae is polyphyletic. They have arisen from non-coastal ancestors among eight subfamilies. Furthermore, many coastal species belong to genera that include non-coastal species. Thus, we should expect a diversity of structural, physiological, and behavioral adaptations among them.

### The Pacific ocean as the main evolutionary center

Selecting only those genera that are exclusively coastal and separating them into three groups according to their provenance, yields the following:

Coastal genera from the Pacific only: 40

From the Pacific and elsewhere: 14

From anywhere except the Pacific: 10

The Pacific Ocean (including its various seas such as the East Sea and South China Sea) is clearly the major cradle of coastal Staphylinidae.

Coastal staphylinids have arisen within all four major lineages of the family, and even within these lineages they are polyphyletic. The simplest explanation is that the Pacific is oldest ocean (having arisen from the ancient ocean called Panthalassa). An alternative explanation might be that the shores of the Pacific are longer than the shores of other oceans, and somehow the species/area relationship (which has generally been supported for the staphylinid fauna of land masses) would suggest greater diversity on Pacific shores. A second alternative might be that non-coastal staphylinid faunas in lands surrounding the Pacific are more diverse than elsewhere and somehow had greater genetic plasticity. A third alternative might be that somehow the Pacific coasts have or had a special abundance of suitable habitat.

The ten genera not represented in the Pacific are: European (*Actocharis*, *Brundinia*, *Pseudopasilia*, and *Phytosus*), far South Atlantic (*Chilodera* and *Acticola*), Caribbean (*Briaraxis*), Gulf of Mexico (*Ophioomma*), and South Atlantic and Indian Ocean (*Pareiobledius* and *Chetocephalus*). *Briaraxis*, *Chilodera*, *Acticola* and *Ophioomma* are monotypic and because there are few specimens with habitat information, it is not certain whether the latter three are truly coastal. Europe, with four genera in the North Atlantic Ocean (and/or Mediterranean, Black, North, Irish, and Baltic seas) falls second to the Pacific Ocean in terms of precinctive genera.

*Actocharis* and *Phytosus* are currently placed in the tribe Phytosini. This tribe includes two other genera that have representative species in the Pacific. Unfortunately, this tribe requires revision and its monophyly is uncertain. Therefore, it is not clear whether *Actocharis* and *Phytosus* evolved from coastal ancestors that immigrated from the Pacific, or whether they evolved from non-coastal European ancestors. The discovery of a species of *Phytosus* in the Caribbean is discussed later.

Apart from whether *Chilodera* and *Acticola* are truly coastal, their attribution to the South Atlantic Ocean needs qualificationbecause they are known only from the Falkland Islands. Could they have a yet undetected distribution elsewhere? Another coastal genus, *Crymus*, has two species, distributed in the South Atlantic Ocean (Falkland Islands and South Georgia) and South Pacific Ocean (the Campbell and Auckland Islands of New Zealand) thus having a sub-Antarctic distribution. None of these genera extends farther north. Antarctic seas do not seem to have provided a conduit for migration of less cold-hardy coastal staphylinids around Cape Horn or the Cape of Good Hope. Forty genera remain confined to the Pacific and have not been detected in the South Atlantic. Among the 14 genera that are known from the Pacific and elsewhere, only two (*Halobrecta* and *Diglotta*) have species in the South Atlantic: *Halobrecta algophila*, which may have arrived adventively via shipping activities, and *Diglotta brasiliensis*.

Even the Arctic Ocean has a partially coastal species (*Micralymma brevilingue*) whose congener, *Micralymma marina*, is truly coastal and extends south through the Atlantic Ocean, east to the North and Baltic seas, and west to New England and Atlantic Canada. A third species (*Micralymma laticolle*), also from the Arctic Ocean, does not belong to *Micralymma* (seeHabits, Habitats, and Classificatory Notes), and should be transferred to another genus. A fourth species, in Eurasia, is not coastal.

The foregoing discussion suggests that almost all coastal genera of Staphylinidae evolved from non-coastal ancestors on the shores of the Pacific Ocean. Some apparently dispersed by migrating to the shores of other oceans. Geological constraints are outlined below.

The primordial ocean surrounding Pangaea in the Permian was Panthalassa, generally accepted as the direct antecedent of the Pacific Ocean (Scotese 2010). The breakup of Pangaea into Laurasia and Gondwanaland began by formation of the Tethys Sea which was the antecedent of the Indian Ocean and the Red Sea. Earlier authors (1960s) included the Mediterranean Sea, but recent authors credit the Mediterranean as being a separate entity, perhaps part of the Neotethys ocean basin, formed ca. 200 mya. The separation of Laurasia continued during the Triassic with the beginning of the North Atlantic Ocean. In the Jurassic, the North Atlantic Ocean widened and the Tethys Sea began to narrow. Not until the Cretaceous did the South Atlantic Ocean began to widen. In the Eocene, the South Atlantic Ocean became connected to the South Pacific Ocean by opening of the Drake Passage between Antarctica and South America some 41 mya (Scher and Martin 2006). The Mediterranean Sea lost its connection to the Indian Ocean some 15 mya. About 7 mya, the Mediterranean lost its narrow connections to the North Atlantic, its waters evaporated and it became a salt desert. At 4.86–4.6 mya, the Strait of Gibraltar opened, and water poured in from the North Atlantic in an immense flood (Garcia-Castellanos et al. 2009). The North Atlantic Ocean lost a major connection to the Pacific Ocean by completion of the Central American isthmus 3.5–3.1 mya (Bartoli et al. 2005). This is the geological background against which the current distribution of coastal Staphylinidae may be understood. It suggests that the Tethys Sea (and its extension, the Neotethys ocean basin), could have offered an early conduit from the Pacific via the Mediterranean to the Atlantic until the connection between the Mediterranean and Indian Ocean was lost, some 15 mya. However, between 7 and 4.8 mya, the Mediterranean became a salt desert and surely lost those parts of its coastal fauna that could not tolerate extreme salinity and aridity; the coastal Staphylinidae that had dispersed to the Atlantic could have survived, but other populations would have become extinct within the Mediterranean. [This account would to some extent be altered if recent ideas of a closed Pacific Ocean in the upper Triassic to lower Jurassic (McCarthy 2003) become generally accepted, although those ideas may make current distribution of Pacific Staphylinidae easier to explain].

### The width of the north Pacific ocean has not been a severe barrier

Liparocephalini (Aleocharinae) include seven genera containing 24 species, and all species are coastal. Five genera inhabit the North Pacific Ocean only, and four of them have representatives on both sides of it (i.e., in Asia and in North America). However, only two of the 24 species, *Diaulota aokii* and *Paramblopusa borealis*, occur on the shores of both continents. This suggests that the width of the North Pacific Ocean has not been a severe barrier to dispersal, and that most dispersal occurred in the remote past, allowing subsequent speciation.

Ahn et al. (2010) showed that the distribution of Liparocephalini was congruent with geological history, including the splitting of Pangaea and the isolation of the Palearctic from the Nearctic. They hypothesized that the ancestor of the tribe was distributed along the Panthalassan Ocean and repeated dispersals occurred into the Nearctic from the Palearctic.

Although these insects, presumably with favorable currents and winds, may cross oceans, we must suspect that barriers are formed by intervening land masses. This suspicion is heightened by dearth of records from inland localities, with just a few from California’s Salton Sea (which once was connected to the Gulf of California). Thus, the biogeography of coastal staphylinids differs greatly from that of non-coastal species.

### Westward dispersal across the Atlantic to the Caribbean

*Phytosus* (Aleocharinae: Phytosini) has five European species, the range of two of which includes the Canary Islands; their presence on these islands suggests an ability to disperse. Three other species are known: one from the Azores, one from Senegal, and one from Guadeloupe in the Caribbean. Their ancestors are postulated here to have dispersed from Europe in the remote past. The westward dispersal of the putative ancestor of *Phytosus caribeanus* across the Atlantic at the latitude of the Caribbean could have been assisted by trade winds.

*Heterota* (Aleocharinae: Homalotini) contains three species in the Pacific, three in the Indian Ocean, two in the Red Sea, and one in the Mediterranean. A tenth species (*Heterota plumbea*) inhabits the Atlantic coasts of Europe, the Mediterranean, and the Canary Islands. It has also been found in the Caribbean (Jamaica and the Yucatan peninsula of Mexico) and Florida (Frank and Thomas 1984b). Small winged insects adapted to a seashore existence could be prime candidates for transoceanic dispersal by the prevailing winds such as from southern Europe or northern Africa to the Canary Islands and thence to the Caribbean.

### Northward dispersal from the Red sea to the Mediterranean

The Red Sea may have been a conduit for dispersal from the Indian Ocean to the Mediterranean. If such dispersal did occur, a subsidiary question is whether it occurred recently, after the building of the Suez Canal, whether it occurred long before that and east or west of the Sinai Peninsula, or whether it occurred in the remote past before sea connections between the Red and Mediterranean seas closed ~15 mya. In this context it is worth noting that there is no evidence for dispersal of coastal staphylinids from the Indian Ocean via the South Atlantic to the North Atlantic.

*Bryothinusa peyerimhoffi* (Aleocharinae: Myllaenini) is reported from the Red and Mediterranean seas.

*Heterota* (Aleocharinae: Homalotini) has three species in the Pacific, three in the Indian Ocean, two in the Red Sea, and one in the Mediterranean (and one, previously mentioned, distributed from the Mediterranean to the Caribbean). Current distribution suggests that the Red Sea may have been an ancient conduit to the Mediterranean.

*Cameronium* (Aleocharinae: Homalotini) contains three species in the Indian Ocean, one in the Red Sea, and one (*Cameronium liebmanni*, not listed) at inland localities in Algeria, adjacent to the Mediterranean. Current distribution suggests that the Red Sea may have been an ancient conduit to the Mediterranean. Could *Cameronium liebmanni* have found refuge at inland locations as the Mediterranean Sea became a salt desert ca. 7 mya? A species from the Gulf of California was attributed to this genus by Moore (1964a), but this assignment needs reassessment.

### Eastward dispersal from the Pacific to the Atlantic

*Cafius* subgenus *Euremus* contains 10 species in the Pacific and a few others elsewhere. Because *Cafius bistriatus* has one subspecies (*Cafius bistriatus bistriatus*) on the Caribbean and Atlantic coasts of North America, and one subspecies (*Cafius bistriatus fulgens*) in the Gulf of California and on the Pacific shores of California and Baja California, it has been posited that it and the ancestor of *Cafius rufifrons* arrived in the Caribbean from the Pacific (Frank et al. 1986). If Central America is now an insuperable barrier to such dispersal, then the date of dispersal was probably before the connection of Central America to South America 3.5–3.1 mya.

*Aleochara litoralis* (Aleocharinae: Aleocharini) occurs on the Atlantic coast of North America from Florida north to Newfoundland and Quebec, as well as on the Pacific coast from California north to Alaska. There has been no report of specimens from the Canadian Arctic that could explain a natural dispersal.

### The distribution of coastal species of non-coastal genera

Selecting the coastal species belonging to non-coastal genera and placing them into the same categories as in preceding list yields the following:

Coastal species of non-coastal genera from the Pacific only: 10 genera, 39 species

From the Pacific and elsewhere: 3 genera, 4 species

From anywhere except the Pacific: 13 genera, 35 species

The genus *Bledius* is excluded from this compendium.

Here, it can be seen that the numbers of genera and species from the Pacific only are almost identical to those from anywhere except the Pacific. The species included are such as *Philonthus nudus* and *Heterothops asperatus* (Pacific Ocean), *Medon pocoferus* (Mediterranean), *Medon marinus* (Indian Ocean), *Medon prolixus* (Japan), *Sunius ferrugineus* (Caribbean), and *Sunius minutus* (United States). They belong to genera that have many non-coastal species. Although they have evolved to occupy coastal habitats, their anatomical characters do not set them apart as belonging to genera that are distinct from the non-coastal ancestors. In other words, their adaptation to coastal habitats is probably more recent than in genera that consist only of coastal species.

### The genus Bledius is a special case

We do not include the genus *Bledius* in the above accounts because we believe it is a special case, with possibilities of overland dispersal during its evolution.

### Effects caused by humans

There can be few species that have not been affected by human activity, and coastal staphylinids are no exception. *Teropalpus unicolor* is native to New Zealand and now occurs adventively in Australia, South Africa, on the Pacific coast of North America, and the south and southwest coasts of Britain (Hammond 2000). *Adota maritima* (syn. *Atheta immigrans*)is known from the Pacific coast of North America and adventively from Britain (Easton 1971, Hammond 2000). The European *Halobrecta flavipes* is another likelycandidate for a list of species dispersed by shipping because it has been detected on the coasts of Chile, Australia, and eastern Canada. Another European species recently detected on the Atlantic coast of Canada is *Diglotta mersa* (Klimaszewski et al. 2008).

The building of seawalls for construction of marinas and docks, and for protection of buildings from wave action, damages beaches and in some places may harm coastal staphylinid populations. The senior author remembers that drifted seaweed accumulated on a beach by the west end of a bridge at Ft. Pierce, Florida in the early 1970s and harbored *Cafius* species. Twenty years later, the area had become a marina with seawalls and without seaweed accumulations; very many undocumented changes are likely to have been caused by similar constructions elsewhere. Human enjoyment of beaches to some extent depends upon the cleanliness of the beaches, and humans have employed beach-sweeping machines to remove not only garbage but also ‘unsightly’ drifted marine algae, which are the habitat and basis for food chains for some coastal staphylinids (Frank et al. 1986). An unusual sign at a public beach at Santa Barbara, California in summer 2009 (seen by the senior author) explained that seaweed was not being cleared there because it was the habitat of invertebrate animals that serve as food for birds. Oil spills on beaches are surely harmful to staphylinid populations, although we have seen no documentation. The compaction of beaches by human activity (vehicular as well as crowds of people) may be detrimental to Staphylinidae. Recently much-discussed threats to coasts and oceans are a rise in sea level and acidification, but their potential effect on coastal staphylinid populations have not been discussed in print if at all.

## Summary

1. Not all Staphylinidae found on seashores are coastal.

2. Coastal Staphylinidae comprise <1% of all species of Staphylinidae.

3. Coastal Staphylinidae have arisen in eight of the subfamilies, are polyphyletic, and have diverse adaptations of structure, physiology, and behavior that allow them to exist in such habitats.

4. There are far more strictly coastal genera in the Pacific than elsewhere and the Pacific appears to be the most important evolutionary center for this group.

5. There are approximately as many coastal species (belonging to otherwise non-coastal genera) in the rest of the world as there are in the Pacific.
